# Perspectives of adolescents from low-income households in Berlin—nutrition described by food affordability, household routines and autonomy: results from an exploratory qualitatively-driven mixed-methods study of cases

**DOI:** 10.3389/fpubh.2026.1791106

**Published:** 2026-08-28

**Authors:** Sarah B. Blakeslee, Anne Schirmaier, Lara Steyer, Steven Ngandeu Schepanski, Georg Seifert, Wiebke Stritter, Gonza B. Ngoumou

**Affiliations:** 1 Charité Competence Center for Traditional and Integrative Medicine (CCCTIM), Charité—Universitätsmedizin Berlin Corporate Member of Freie Universität Berlin and Humboldt Universität zu Berlin, Berlin, Germany; 2 Department of Pediatric Oncology and Hematology, Charité—Universitätsmedizin Berlin, Corporate Member of Freie Universität Berlin and Humboldt Universität zu Berlin, Berlin, Germany

**Keywords:** adolescent nutrition, adolescents, dietary practices, low-income households with children, mixed-methods research, socioeconomic inequality, youth perspectives

## Abstract

**Introduction:**

Food insecurity threatens the nutritional intake of young people, including their health and well-being. It is known that the link between nutrition and low income exacerbates socioeconomic disparities. In Germany, low-income families are entitled to subsidies, but evidence suggests that financial assistance is insufficient for an adequate diet. Young people from low-income families are affected by this, but little is known about their experiences.

**Materials and methods:**

A two stage, qualitatively-driven, exploratory, mixed-method study with consecutive design was undertaken with young people (14–17 years) from low-income households in Berlin. Photo-documentation, interviews and participant observation were conducted, and quantitative data was gathered on nutrition, health, wellbeing and financial situation of young people and their families. Data types were analyzed thematically and descriptively, then reported as cases.

**Results:**

In total, six young participant cases (5 female, 1 male), with a median family weekly food expenditure of 150 € completed the first stage of the study and most rated health and nutrition as important. Recruitment was very challenging, and only one of the participant completed all parts of the second stage. Therefore, additionally three youth experts were interviewed. In the narratives of the young people two important and distinctive settings for consuming food emerged: (1) eating within the family setting versus (2) external food consumption. Both in interviews and family’s meal observations, the daily organization, preferences and practices were guided by parents and mostly accepted by the young people within the home. Observed shopping trips and interview narratives about finances underscored awareness of the young people about family finances. School was not considered a conducive environment for finding nutritious and affordable food options. Friends, school peers and social media trends influenced consumption habits. Young people developed strategies to access desired foods that became part of their developing autonomy.

**Conclusion:**

The study provides valuable and underrepresented insights into the food environments of young people from low-income households in Berlin with an integrated methodological approach, showing how their everyday experiences with food, health and nutrition centered on the home and independence articulated among peers. Lack of access to healthy, affordable food at school underscores income discrepancies among pupils.

**Clinical trial registration:**

https://drks.de/search/de/trial/DRKS00031853 Identifier DRKS00031853.

## 1 Introduction

Food security, as defined by the United Nations Food and Agriculture Organization (FAO), exists when all people have physical, social and economic access to sufficient, safe and nutritious food that meets their dietary and psychological needs for an active and healthy life at all times (1). Particularly for children and adolescents, adequate nutrition is essential for physical growth, cognitive development, and long-term health and well-being (2). Conversely, food insecurity is marked by the absence of these elements and often associated with low and middle-income countries in its severest form (3). This is rooted in the theoretical frameworks of the socio-ecological model that emphasize the influences of social and environmental factors affecting children and grounded in the social determinants of health (4, 5). However, a lack of sufficient access to food is increasingly recognized as a relevant issue in high-income countries, like Germany, one of the most economically developed countries in the European Union (6–8).

In Germany, resulting diets are frequently characterized by an imbalance between energy density and nutrient adequacy, resulting in a mismatch with nutritional recommendations and increasing the risk for both undernutrition and overnutrition (9). In this context, placing a particular focus on populations with lower socioeconomic status (SES) is important, since food choices are deeply intertwined with social and economic determinants (6, 10, 11). The contemporary food environment theory underscores the way that forming nutritional discrepancies may stem from and are embedded in social practices such as material deprivation, household resources, family routines, peer and school contexts, neighborhood infrastructures, food availability, affordability, cultural norms, and policy conditions (7, 8).

Despite redistributive measures such as taxation and social transfers, 21.1% of the German population were at risk of poverty or social exclusion in 2024, with children and adolescents disproportionately affected at 22.9% (12) and children and youth with immigration backgrounds are four times more likely to be at risk (13). Notably, rates of relative income poverty are consistently higher in Berlin compared to the national average (14). Families with limited financial resources are entitled to state subsidies such as *Bürgergeld*^1^ or supplementary benefits. The social support is supposed to cover essential needs (15). However, the ability of this support to cover basic nutritional needs has been subject to critical debate (16–18). It has been demonstrated that available state subsidies are generally too low to cover the actual food costs of children and adolescents, regardless of the type of their dietary pattern and despite additional subsidies for school and daycare lunches (16). Generally in Germany, people affected by lower income tend to have unhealthier diets than people with a higher income (19). Findings from the nationwide German Health Interview and Examination Survey of Children and Adolescents (KiGGS, 2014–2017) confirmed that children from low-income households consume less fruits and vegetables than their more affluent peers, and are more likely to experience health impairments, and psychological distress (20). Young people from households in the lowest income bracket show psychological problems more often compared to those from households with higher income ranges (21). The social gradient in health is further reflected in poorer self-rated health, lower life satisfaction, and reduced physical activity among adolescents from low socioeconomic backgrounds (22). Health and nutritional deficiencies, especially for fruit and vegetable consumption and physical exercise for the lowest socioeconomic bracket are, not applicable to Germany alone but mirrored internationally (23). For instance, studies in the United States have shown that the necessary caloric intake is impeded both by less income available for food and also a higher cost per calorie for more nutritious fruits and vegetables (24).

Despite this body of evidence, the lived experiences and perspectives of young people and adolescents from low-income families have received limited attention in research. Children and adolescents are active social actors who interpret, negotiate, and respond to these conditions in their everyday lives, consistent with child participation perspectives that emphasize young people’s right to express views on matters affecting them (25) Previous studies have predominantly focused on the parental perspective, often overlooking the voices of young people themselves. Qualitative findings from Pfeiffer et al. (26) indicate that food insecurity in Germany is associated with both nutritional deficiency and social exclusion, with families employing diverse coping strategies to compensate for deficits, such as skipping meals, buying discounted food, and reducing dietary quality and diversity. The Giessen Nutrition Study on the nutritional behavior of poor households (GESA-study), which explored the daily lives of low-income families in a socially disadvantaged urban district, revealed overlapping needs. This study found that nutrition was often given a lower priority over more urgent demands such as childcare, isolation, or conflict. Financial constraints led to income being stretched over weeks, especially toward the end of the month, which was characterized by monotonous diets and, in some cases, hunger (27). Furthermore, the mixed-method, multidisciplinary health and nutritional status of persons living in households at risk of poverty with children in Germany (MEGA_kids) study emphasized the central role of food prices as a barrier to healthy eating in poverty-affected households. Reported coping strategies included purchasing discounted food, cutting back in other areas, and compromising on the quality, quantity, and variety of meals (28).

To date, little is known about how young people growing up in poverty perceive their nutritional environment or how they navigate daily food-related challenges. This gap is particularly striking given that subjective perceptions play a key role in shaping health behaviors and dietary decisions during adolescence. While some international studies have engaged children directly (e.g., 97, 98), comparable data is lacking in Germany, and specifically in Berlin, where poverty rates are highest.

Understanding how children and adolescents from low-income households perceive their nutritional situation is vital to developing responsive, needs-based interventions and policies. Their perspectives can provide critical insights into barriers and facilitators of healthy eating, inform the design of targeted support measures, and ensure that health promotion strategies are grounded in the lived realities of those most affected. By centering young people’s own accounts, this approach enables a nuanced understanding of perceived barriers and facilitators of healthy eating and generates practice-relevant knowledge for targeted, needs-based health promotion. For this reason, an exploratory design with a qualitatively-driven methodology was chosed to, investigate the subjective perception and food environment of young people from low-income families in Berlin with the E-Kids study.

## 2 Materials and methods

The E-Kids project aimed to better understand the experiences of young people living in low income families regarding their dietary perspectives and preferences. This study was registered in the German Clinical Trials Register in May, 2023 (DRKS00031853) and granted approval by the ethical commission of the Charité—Universitätsmedizin Berlin (Nr. EA2/025/23). Reporting followed the Consolidated Criteria for Reporting Qualitative Research (COREQ) (29). The following questions were the focus of this investigation:

1. How do adolescents affected by poverty, as well as their families, describe their living situation with regard to nutrition and food security?
2. How do these adolescents and their guardians assess the young people’s health?
3. What kind of support for their nutrition do adolescents want and need?

### 2.1 Study design

**Figure 1:**
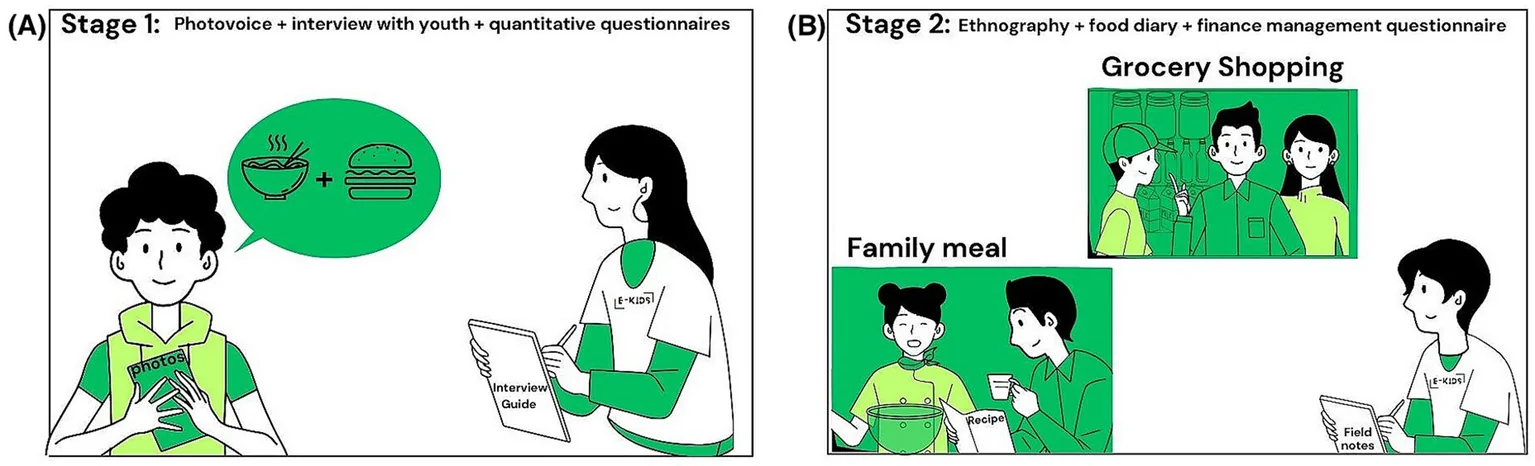
E-Kids project design. The E-Kids study was conducted in two stages. The first stage **(A)** was mandatory for participants who voluntarily consented to participate and included a photovoice by the adolescents, an interview with the adolescents and questionnaires on health, nutrition and self-efficacy. Stage two **(B)** was optional and included an ethnography, food diary and a finance management questionnaire.

**Figure 2:**
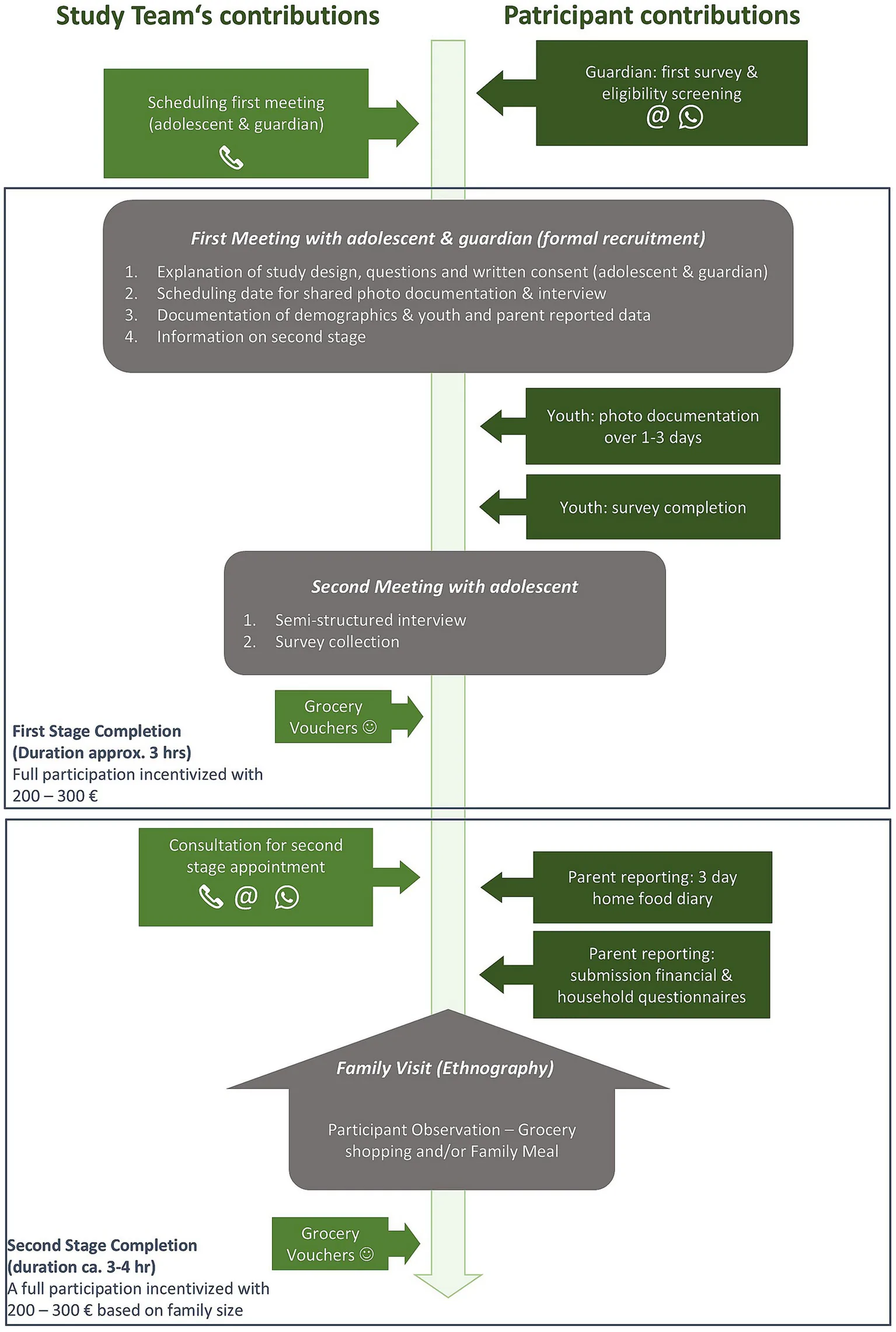
E-Kids data collection process. During the two E-Kids study stages, data was collected during a total of approximately 3 h during the first stage at two meeting time points. Families were incentivized to complete this stage with 200–300 € of grocery store vouchers based on family size after data completion. The first meeting was held with adolescents and parents and during this meeting the following information and data was planned and collected: written consent, scheduled date of photo documentation and interviews, documentation of demographics (age, sex, BMI, number of household members, monthly income, residential district, parent degree, school form, employment, weekly nutritional costs), and information on the second stage was given. At the second meeting during the first stage, a semi-structured interview was conducted during which the sent photo documentation was discussed, and the surveys were completed (nutrition and health adapted from the Boles questionnaire, self-efficacy questionnaire). Participation in the second stage was voluntary and consisted of additional survey completion (3-day food diary, financial management questionnaire) and a scheduled participant observation of a family meal and/or grocery shopping trip over a total of approximately 3–4 h and incentivized by 200–300 € additional grocery store vouchers based on family size after data completion.

The research objective of E-Kids was to preliminarily explore nutritional experiences directly with young people from lower income families. Using an exploratory, two-stage, mixed-methods study design of cases, we collected multi-layered data from young people and their families (see Figures 1, 2). Young people photo-documented images of their consumed food, participated in interviews and took part in participant observations. We explored the young people’s own opinions of their physical wellbeing and how they perceived their own nutritional health along with their needs, desired support, and wishes regarding their nutritional health. To give greater context to the qualitative data and to understand these within the context of the families, we asked young people and their parents or guardians to answer quantitative questionnaires on nutrition, health, wellbeing and their financial situation. Additionally, in order to gain additional insight into the interviews and observations conducted with young people and their families, expert interviews were conducted with staff from youth centers and other social workers and gave additional perspectives and context of those working directly with these youth. Analyzed and triangulated data was synthesized and reported post-hoc with a pragmatically-adapted as case analysis (30, 31). A study protocol is available (see Supplementary 1.1 Study Protocol).

### 2.2 Recruitment

Our eligibility criteria sought a purposeful case sample of young people 14–17 years of age living in Berlin with a consent form signed by their parents or legal guardians, in a family with at least one parent (legal guardian) receiving income-based government subsidies, sufficient knowledge of German, willingness to participate in at least one interview, to document photographs of meals, and to complete questionnaires during stage one of the study. Within the German context, the subsidies are applicable for individuals and families who are no longer covered by unemployment insurance and have broad coverage for both German and non-German populations residing in Germany, who were eligible for the study regardless of citizenship or immigration status (15). Individuals with a diagnosis or participation in another study that could limit participation were excluded.

Recruitment is challenging for this demographic, and multiple strategies with demonstrated success were implemented, such as partnerships with community groups, snowball sampling of contacts and strategic use of community gatekeepers (32). Recruitment of youths was facilitated through flyers distributed at 130 pediatric practices, secondary schools as well as through in-person presentations at 10 neighborhood youth centers, 4 community centers, 3 food banks and 2 administrative centers. Community gatekeepers coordinated with researchers (AS, a nutrition and public health researcher, LS, a female doctoral student) helped ensure a diverse sample from neighborhoods with low socio-economic populations (33). A purposeful sampling strategy was used for recruiting specifically targeted cases (34), whereby initial information was collected and analyzed regarding of young people’s age range, place of residency, family background, gender and availability. After initial contact (LS), potential participants were assessed for eligibility using a contact survey. Following the initial step, recruitment strategies were then adjusted to target maximum variation in gender, age, backgrounds and school form attendance. However, given the exploratory nature of the study, we did not anticipate reaching theoretical saturation.

Parents or caregivers of eligible youth were contacted and scheduled an initial meeting with a researcher (AS & LS) to explain the study details and background, provide written information and gather written consent from the youth and guardian. Participants consented to a two-stage research process of which only the first stage was required for participation (see Figure 1). Participants were given financial incentive in the form of supermarket vouchers according to the family size after completing stage one of the study. Additional incentives were given for stage 2 completion (see Supplementary 1.2: incentive scale).

After encountering recruitment challenges, we adapted our study approach during the recruitment phase. In particular, recognizing the extensive experience that the social workers had with young people from low-income families, we decided to include their perspectives to better understand barriers to participation and gain their insights on nutrition activities with low-income adolescents. To this end, we recruited a purposeful sample (34) of social workers from local neighborhood-based organizations offering programs and support to low-income youth and their families. These social workers consented to a semi-structured expert interviews providing input and sharing their experiences of working with youth from low-income households and nutrition-based activities. Their perspectives helped us to hone our recruitment strategy of cases and present a triangulated perspective to the small sample we were able to gain for our study.

### 2.3 Data collection and analysis

#### 2.3.1 Stage one

After giving written consent, participants took part in stage one of the study. In stage one, youth were asked to document and photograph their meals over 1–3 days via smartphone and share this with the research team (LS). After documenting meals through this pragmatically-adapted photovoice method (35, 36), the young people were interviewed in an agreed-upon location chosen by the participant by a female doctoral student (LS) trained in qualitative interviewing and participant observation for this age group. The semi-structured qualitative guideline was developed by formulating questions based on research topics using the SPSS guideline development by Helfferich (37) and adapted to strategies for developmental, cognitive and interview articulation practices for this age group and background. After refining the guideline into central topics, the guideline was pilot-tested in an interview with an adolescent and made adaptions to the guideline be more conversive after several interviews after discussion in the team (LS, AS, WS, SB, GN). This allowed for more closed answers that then permitted greater dialogic flexibility with the aim to elicit more information and reduce power barriers—due to age, status, economic means—between the researchers and participant (38–42) (see Supplementary 1.3: youth interview guideline).

At this stage, youth were also asked to complete a first set of quantitative questionnaires including (a) the attitude of participants towards nutrition and health adapted from the questionnaires by Boles et al., including only the primary 3 questions asking to rate own health as well as importance attributed to health and nutrition on Likert scales from 1 (very good/very important) to 5 (very bad/not at all important) (43) and (b) the general self-efficacy (not nutrition related) captured by the questionnaire of Jerusalem Schwarzer (44). Additionally, basic demographic data were collected from parents/caregivers which included age, sex, height, weight, body mass index (BMI), household, living situation, residential district (classified according to the Overall Index of Social Inequality 2023, which assigned values from 1 to 4). A 12 possible score with four points of social status that are indicated by a high (1), mid-range (2), low (4) and very low (5) social status and 3 levels of dynamic momentum (positive, stable and negative) (45), school form, school degree and employment of both parents, monthly income category and weekly nutrition costs. BMI is displayed as standard deviation score (SDS). BMI-SDS is an age- and sex-adjusted measure of body mass index that expresses a child’s BMI relative to a reference population. It indicates how many standard deviations an individual’s BMI lies above or below the population mean and allows comparison across different ages and between sexes.

Additionally, social workers who consented to participate in expert interviews were asked about their work with young people, observed behavior around nutritional activities and how the study might encourage participation (see Supplementary 1.4: expert interview guideline).

#### 2.3.2 Stage two

After completing stage one, families were asked if they would like to take part in stage two of the study requiring the completion of a 3-day food diary, financial management questionnaire and ethnographical participant observation at a family meal or grocery shopping outing (LS).

In participant-observation using focused ethnography (46), fieldnotes were taken, then digitalized on a field-tested form (see Supplementary 1.5: participant observation fieldnote form).

During stage two, the parents/caregivers who participated completed a five item scale assessing financial management behaviors based on the instrument in Gundersen and Garasky (47). Parents/caregivers reported how often they engaged in practices such as checking bills for accuracy, paying bills on time, avoiding charges, budgeting, and reviewing income and expenses before major purchases. Responses were rated on a scale from 0 to 5, with higher values reflecting more frequent use of recommended financial behaviors. Only the answers 4 and 5 were considered affirmative responses. A financial index was then calculated by adding the number of affirmative responses resulting in an index between 0 and 5. Additional two items captured subjective financial competence. Respondents were asked to rate their confidence in managing household finances (from 1 to 3, 1 = very competent, 3 = not at all competent) and their perceived capacity to handle available resources (from 1 to 5, 1 = very good, 5 = very bad) (47). All the questions were carefully translated into German by a native English speaker (SB, post-doctoral public health researcher and methodological study lead) and slightly adapted.

Parents/caregivers were also asked to rate the health status of their youth on a 1–5 Likert scale (1 = very good 5 = very bad).

Additionally, participating parents/caregivers were asked to specify the frequency of home cooking, the frequency of ready-meal cooking and the frequency of outside meals (all three questions rated on the scale: twice per day or more, once per day, minimum every 2 days, 3 times per week, almost never).

#### 2.3.3 Qualitative analysis

Analysis was guided by the central research questions and conducted as a qualitative content analysis following Kuckartz (48) and underpinned by a co-constructivist epistemology (49) under the assumption that knowledge is co-produced by the interaction between researcher and participant.

Using a deductive-inductive approach to coding, all interviews were audio-recorded, auto transcribed using f4x (50), corrected and anonymized. All qualitative data (photos, interview transcripts and participant observation fieldnotes) were imported and managed into the qualitative analysis software MAXQDA (51). After the interviews were conducted (LS) as a first was sensitization to interview themes, post-interview summaries were written (LS) and these were read and interview audios were listened to by a senior researcher (SB). Interviews of youth and expert interviews were fully coded within a single MAXQDA project. The main themes were first deductively, then inductively coded by one researcher (LS). Then, themes and developing categories were collaboratively discussed after each interview between two researchers (LS & SB) (48, 49). First one researcher (LS) established a deductive code structure from the interview guideline, then coded inductively until a defined coding tree was developed and defined from the interview transcripts. Then these were compared and contrasted in the interviews with the adolescents, then each of the main themes was discussed extensively with two authors (LS & SB) and where needed for analytical differentiation, a third or fourth author was consulted (AS & GN, pediatrician, post-doctoral nutritional researcher, project lead) until agreement was reached among team members in order substantiate interpretation. These main themes of interviews with the adolescents were triangulated with the content of observation fieldnotes, photos and expert interviews, comparing and contrasting until categories emerged and these helped defined the interpretation. Anchor quotes and supporting material (from photos, observation notes and expert interviews) were used to solidify findings (LS) which were then discussed in the wider group (LS, SB, GN, AS) and further validated in a monthly doctoral qualitative analysis meeting. SB, GN and WS (Head of research and experienced qualitative researcher) provided senior guidance throughout data collection and analysis. The triangulated categories provide the main findings to contribute to answering the three research questions.

#### 2.3.4 Integration of quantitative data and synthesis of cases

Quantitative data were analyzed using SPSS statistics [version 29.0.0.0 (241)] and Microsoft Excel (version 1808) to summarize characteristics and key variables. Means, standard deviations and ranges were calculated where applicable. Composite scores were formed for multi-items scales. Given the exploratory nature of the study and its embedding within a qualitative framework, no inferential statistics were performed, and given the predominantly qualitative nature of the data and small sample size, no statistical concept for missing data was deemed necessary. Instead, the data were analyzed with the aim of complementing and enriching the qualitative findings.

The study followed a convergent, qualitatively-driven integration approach (QUAL + quant design), in which different datasets were first analyzed independently and then integrated at the level of interpretation (52). Although the analysis was initially conceived to be descriptively detailed by each type of dataset (quantitative and qualitative data), the minimally available quantitative data was much better suited to an iterative integration of all data. The quantitative data were therefore integrated by comparing findings, identifying convergence, complementarity and divergence and situating data from the quantitative results within qualitative categories (53). Subsequently, all of the available data for each of the young participants in our study were clustered into individual cases for a post-hoc, pragmatic case synthesis, to be analyzed according to the main categories established in the qualitative analysis. The main categories provided the organizational structure for the cases and were augmented by expert interviews in the results. Interviewed social workers gave additional context to case findings, but were not integrated, nor were they linked directly to the individual cases. These steps were performed by SB and GN and supported by the other authors in an iterative process.

## 3 Results

### 3.1 Participant case characteristics

**Figure 3:**
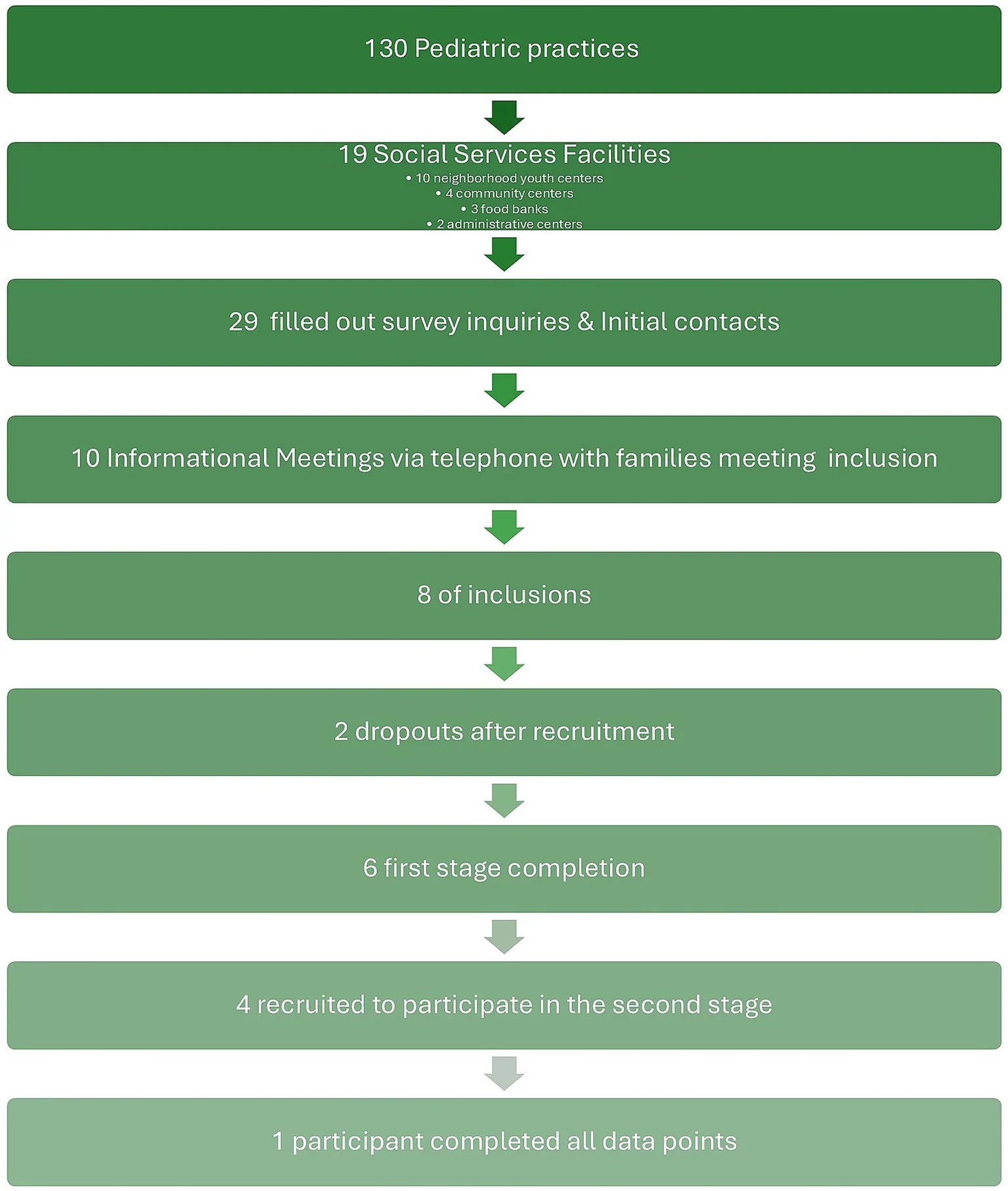
E-Kids recruitment flowchart. Number of facility, adolescent and parent contacts made and participants recruited to the E-Kids study.

Between May 2023 and July 2024, recruitment activities resulted in 29 initial contacts with families and ultimately led to the enrollment of eight participants (Figure 3). Two participants could not be contacted after consenting and were therefore considered dropouts. Six participants completed the first stage, which included interviews, photovoice, and questionnaires. The length of the interviews ranged from 17 to 58 min. One of the interviewed young people was accompanied by an adult chaperone. Of the six participant cases who completed the first stage, four participants also took part in stage two activities, which involved family-based participant observation (one meal situation or one shopping trip), a 3-day food diary, and a finance and cooking habits survey. Complete documentation and responses to all questionnaires for both stages were achieved for a single participant. Additionally, three expert interviews were conducted with a school social worker from a neighborhood youth center (female, between 40 and 50 years. of age, with an educational background in school social work), a youth center coordinator (female, between 30 and 40 years. with an educational background in school social work), and a sports-based social worker (male, between 30 and 40 years. with a technical training background as a sports specialist). The length of the interviews ranged from 38:41 to 1:12:00 min.

### 3.2 Sociodemographic data

**Figure 4:**
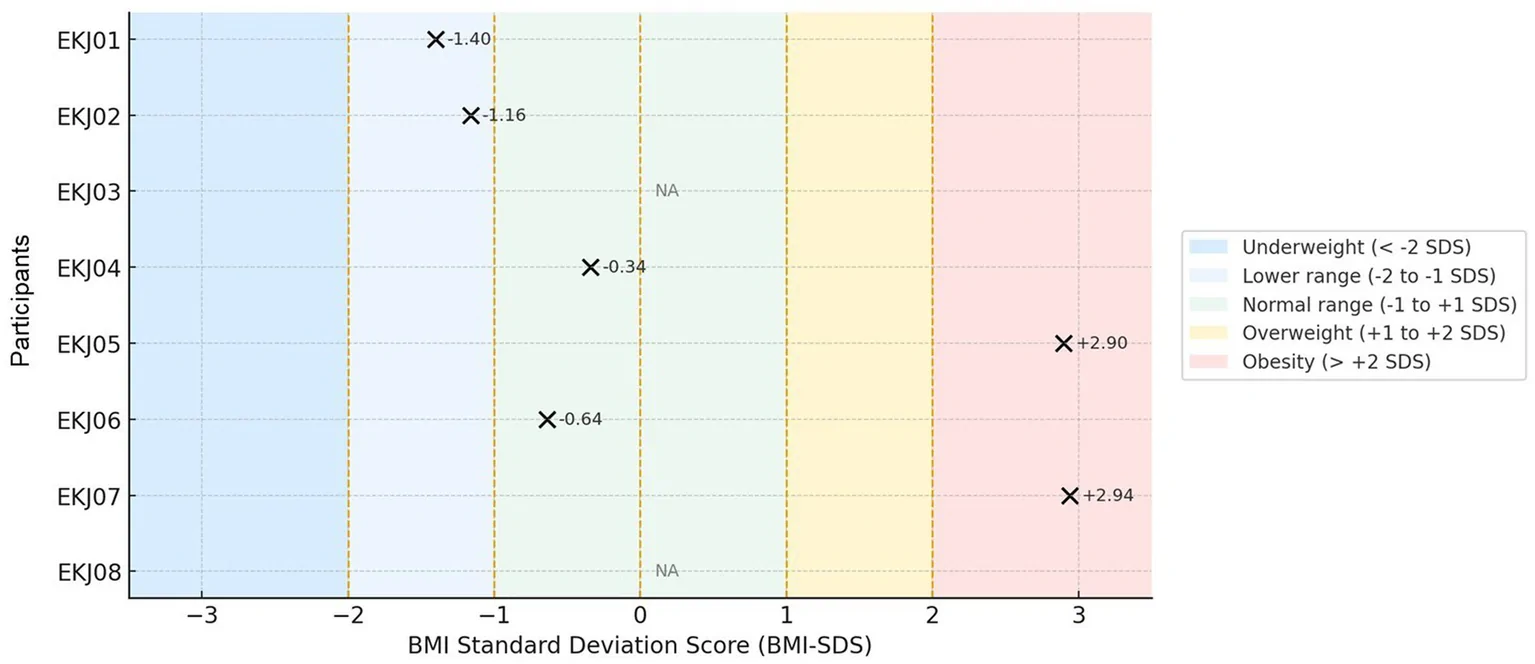
BMI standard deviation scores (BMI-SDS) of study participants in relation to reference ranges. BMI standard deviation scores (BMI-SDS) of study participants (*n* = 8), plotted against reference percentile bands. Each cross represents one participant (IDs anonymized as EKJ01–08), with numeric BMI-SDS values displayed next to the markers. Participants without BMI data (EKJ03, EKJ08) are represented by open circles labeled no data. Shaded background areas indicate reference ranges: light blue = underweight (< −2 SDS), pale blue = lower range (−2 to −1 SDS), green = normal weight (−1 to +1 SDS), yellow = overweight (+1 to +2 SDS), red = obesity (> +2 SDS). Dashed vertical lines mark the SDS cut-offs at −2, −1, 0, +1, and +2. This visualization allows positioning of individual participants relative to international BMI reference standards, accounting for age and sex. Integrated thematic findings.

Table 1 displays demographic data of all youth participant cases. Participants were between 14 and 17 years of age (median 16 years). All but one were female (F = 5, M = 1). BMI values ranged between 18.0 kg/m^2^ (SDS −1.40) and 36.6 kg/m^2^ (SDS +2.94). Four participants were within the normal to slightly lower range, while two participants were in the obese range as shown in Figure 4. According to interview and participation observation data, 5 out of the 6 participants came from families with diverse migration backgrounds from four different countries. The number of family members in the household were between three and seven. Family situations included households with siblings (6/6), living with both parents (4/6) or divorced parents (2/6). Of the principle address recorded for the participant cases, three families lived in mid- to low-status areas, two in mid- to very low-status areas, and one in a low- to very low-status area. Family income ranged between the categories 1,000 € to 1,999 € (2/6), 2000 € to 2,999 € (3/6) and >=3,000 € (1/6) per month. Five out of 6 families spent between 125 € and 200 € (median 150 €) per week for food, one family did not answer this question.

**Table 1:** Sociodemographic and anthropometric characteristics of study participants.

Participant	Sex	Age (years)	BMI	Household size	Residential district social index	School type	Parental education (Parent 1)	Parental education (Parent 2)	Employment status (Parent 1)	Employment status (Parent 2)	Monthly household income	Weekly food costs (€)
EKJ01	Female	17	18.07	5	3–4	IB school (*Gymnasium*)	Lower secondary school (Hauptschule)	Lower secondary school (*Hauptschule*)	Employed part-time	Unemployed/seeking work	€1,000–2,000	0
EKJ02	Female	16	18.03	5	2–4	IB school (*Gymnasium*)	Intermediate secondary school (*Realschule*)	No information provided	Unable to work (disability/health-related)	No information provided	€2,000–3,000	200
EKJ03	–	–	–	–	–	–	–	–	–	–	–	–
EKJ04	Female	16	19.84	4	2–3	IB school (*Gymnasium*)	General university entrance qualification (*Abitur*)	–	Employed part-time	Employed part-time	≥€3,000	200
EKJ05	Female	15	34.60	4	2–3	Secondary school (*Sekundarschule*)	–	–	Employed part-time	Employed part-time	€1,000–2,000	140
EKJ06	Female	14	18.03	4	2–3	IB school (*Gymnasium*)	Intermediate secondary school (*Realschule*)	Intermediate secondary school (*Realschule*)	Unemployed/seeking work	Employed part-time	€2,000–3,000	125
EKJ07	Male	17	36.63	6	2–4	Vocational school (*Berufsschule*)	Lower secondary school (*Hauptschule*)	No information provided	Employed part-time	No information provided	€2,000–3,000	150
EKJ08	–	–	–	–	–	–	–	–	–	–	–	–

Residential district social index = refers to the official district-level socioeconomic classification in Berlin in 2023 (1 = high, 2 = mid-range, 3 = low, 4 = very low social status) (45). School types: *IB School (Gymnasium)* = academic secondary track; *Intermediate secondary school (Realschule); Lower secondary school (Hauptschule); Secondary school (Sekundarschule); Vocational school (Berufsschule)*. Educational attainment: *Abitur* = general university entrance qualification. Employment categories follow participants’ own reports. – indicates missing or undisclosed information.

While our sample strategy did yield heterogeneity spanning age and experiences, three commonalities in social settings emerged during the interviews in which the adolescents described making decisions about what and where they eat: (1) within the family, (2) at school and (3) with peers including influences from social media (for full coding tree Table 2).

**Table 2:** E-Kids coding tree.

Main category	Sub categories	Definition	Exemplary quote
Eating within the family	Daily meals and organization	Description of the person who organizes and prepares the family meals along with what, when and where the food is consumed.	“My mother usually does the shopping; sometimes my father comes along too. Usually my mother also cooks, unless she has an appointment or something—then my father cooks.” (EKJ01, w, 17, IB school)
	Meal decision making	Description of the primary meal decisionmaker for family meals, especially concerning planning and purchasing of ingredients	“What and how I eat? I‘d say whatever my mother makes. Whatever we‘ve got at the moment.” (EKJ01, w, 17, IB school)
	Meal preparation and consumption	How the decision-making of the meals takes place and is operationalized within the family	“We usually just cook whatever we feel like. We talk it over with our mother and decide what we’re in the mood for.” (EKJ02, w, 16, IB school)
	Home meal preferences	Logistics, financial consideration and connections to home meals	“My favourite food is Persian cuisine. I’m always really happy when I come home and, as soon as you walk in the door, you can already smell it. That just makes me happy.” (EKJ06, w, 14, IB school)
	Nutrition and religious practice	How religion fits into family life and meal consumption and how this is practiced	“It’s just been that way from the start. […] Well, that’s how it is in our family, I’d say, so nobody eats pork.” (EKJ01, w, 16, IB school)
	Healthy eating and nutritional adequacy	Understanding of own nutrition and consumption and ways that this is described or practiced	“There was definitely some healthy food, but of course there were also things like wraps with Nutella—which obviously isn’t healthy. But the teachers do make an effort to address these things with the younger kids. With the older ones, though, you really don’t have any influence anymore.” (EKJ01, w, 17, IB school)
Eating at school	Availability and quality of school food	Descriptions of food consumption during school hours and what food is eaten during the school day	“There is a cafeteria, but they only serve waffles or sweet buns or things like that.” (EKJ04, w, 16, IB school)
	Relevance to nutritional adequacy	Description of what portion of daily intake is consumed during the school day	“If I only have school until 1 p.m., then I don’t take anything with me to school. (…) If I have school until 4:30 p.m., I really always go and get something to eat.” (EKJ04, w, 16, IB school)
Eating with peers	Influence from friends	Descriptions of foods that peers and friends are interested in and how interactions around these foods take place	“Yes, but [Takis] are expensive. So we just shared them.” (EKJ02, w, 16, IB school)
	Eating during free time, hobbies and activities	Descriptions of how food is consumed during other activities/hobbies outside the home	“Well, I only hang out with my friends at school and after school. But I don’t go out with girlfriends to eat or anything like that.” (EKJ05, w, 15, secondary school)
	Social media and food trends	Description of what role social media plays in food information and consumption	“So we follow a page on TikTok that always goes to different food places. (…) Then we vote on it and try out different places. I really like that.” (EKJ04, w, 16, IB school)
Financial awareness and deprivation	Awareness of limited resources	How the financial availability of food, ingredients and purchasing power is described	“A lot of what we buy are sale items. Because, to be honest, everything is ridiculously expensive everywhere, and that’s just annoying because at some point you just don’t feel like buying anything anymore.” (EKJ07, m, 17 vocational school)

Main categories provide the study findings, sub-categories correspond to findings within each category. Definitions describe the sub-categories and exemplary quotes provide one anchor quote for each of the sub-categories.

Within the family setting, the participants described food practices shaped by religion, traditions and customs, household routines and parental organization. In contrast, in the setting of school and social interactions with peers, the young people expressed greater independence and spontaneity in their food choices, although financial constraints and the availability and cost of food at school limited these choices in practice. Social media was often linked to peer eating and trends, providing ideas and aspirations for trying new foods.

Across all case settings, two themes emerged as important to food-related opportunities and decisions during adolescence for this group: (1) financial awareness and deprivation, reflected in a clear understanding of limited resources and adaptive decision making around food; and (2) the potential of structural access and trust-based environments, such as youth institutions and school environments, to shape and support access to healthy nutrients.

The quantitative data extended these findings by situating different dimensions of nutrition (self-reported dietary patterns, self-efficacy, self-reported perception of the importance of health and nutrition, as well as self-reported financial management skills by the parents, also see Supplementary 1.6 Parent Questionnaires) within the identified qualitative themes. This was intended to provide a more nuanced understanding of how reported behaviors relate to lived experiences and structural constraints.

Taken together, triangulated case data from interviews, field observations, photovoice submissions, dietary records and questionnaires illustrate the opportunities and the limitations that adolescents in financially constrained households face in meeting their nutritional needs across everyday settings.

### 3.3 Eating within the family setting

#### 3.3.1 Daily meals and organization

Interviews with young people described heterogeneous practices of eating within the family setting. Depending on family size, constellation, number of family members eating together on a regular basis and living situations, practices ranged from regular shared meals to more individual consumption of pre-prepared home meals or purchased groceries consumed within the home (see Supplementary 2.1: photovoice). In all families, the primary person reported and observed to prepare meals was the mother, who arranged their schedule around food preparation. Parent work shifts, such as night shifts, and the young person’s school schedule influenced eating time. Preparation of lunches or food for after school was often done the night before, sometimes prepared by the young people themselves, sometimes as extra portion of family meals.

#### 3.3.2 Meal decision making

Meal planning was reported to be mostly directed by parents or guardians, especially mothers, including what foods were purchased and preparation based on available resources and personal preferences. Adolescents discussed how they coordinated with their parents to prepare food for themselves to take to school.

*“So, I always tell her what I’d like to have for school, because it’s not like she really checks if we have apples, since I'm the only one who eats them.” (adolescent participant EKJ02, female, 16 years, International Baccalaureate (IB) school)*

For the most part, the young people described how they accepted family norms and prepared foods without special requisites.

*"What and how I eat? I‘d say whatever my mother makes. Whatever we‘ve got at the moment." (adolescent participant EKJ01, female, 17 years, IB school)*

However, frustration also appeared when preferences conflicted or clashed with financial limits.

*“Then I tell my mother that we maybe shouldn't eat meat every day, but instead have something without meat. But she says that's not possible because of my father and so on. He can't do without meat and complains when he doesn't get meat.” (adolescent participant EKJ05, female, 15 years, secondary school)*

Some participants mentioned how they tried avoiding conflict by not expressing their preferences, due to family dynamics, or because parents were viewed as the primary authority figure in the family.

#### 3.3.3 Meal preparation and consumption

Most participants described family meals prepared at home as the norm, as opposed to take-out or restaurant meals. One participant described that trying new restaurants occurred only with friends or a financially independent older sibling.

*Well, I don't pay for anything, my brother paid for it. He already has his own family and does his own thing.” (adolescent participant EKJ04, female, 16 years, IB school)*

This pattern was supported by data from field observation and parental questionnaires.

During field observation of shopping trips in two families, adolescents worked together with their parents like a well-rehearsed team. They helped select groceries, pushed the shopping cart or followed the shopping list. Families described strategies to visit different stores, to compare and combine offers, as well as discounts and availabilities. When choosing between similar items, cost and quantity outweighed organic quality.

In both families that completed the parental questionnaire data, a preference for home cooking was reported, while take-away or ordered food was described as rarely or never consumed. In one household (adolescent participant 1 EKJ01), convenience products were rarely or never used; in the other (adolescent participant 5 EKJ05), fresh cooking occurred once daily, combined with some convenience products approximately three times per week (see Supplementary 1.6 Parent Questionnaires).

#### 3.3.4 Home meal preferences and practices

For most, family meals provided a connection to traditions, home and family (see Supplementary 2.1: photovoice). Overall, meals at home were rarely questioned. Youth experts all highlighted the important role that they perceived food to play in families facing financial challenges. One expert (*youth expert 1, female, between 40 and 50 years, social worker neighborhood youth center*) described witnessing some parents providing foods preferred by children, such as sweets, snacks or sugary drinks, in what this youth expert perceived to be a way to compensate for lack of financial resources. The expert explained that they understood the food to create an emotional connection between children and parents that may provide comfort in living situations that may be otherwise less comfortable. Mothers were described by youth experts as making strong efforts to provide hot meals and to bring the family together to eat, even in very crowded living situations.

*“For instance, in refugee families (…) I believe that the mothers, when given the opportunity, make a real effort to maintain their traditional eating habits. There is a strong food culture, and they strive to preserve that tradition.” (youth expert 1, female, between 40 and 50 years, social worker neighborhood youth center)*

All participants were asked about religious practices related to food as part of their family routines and meal planning. One participant addressed the topic without being prompted. Religion was not stressed as an important topic independently, but rather described as part of their daily routine, family practice and culture without special emphasis. This was expressed, for example, in statements about religious food restrictions as an accepted and recognized family normality.

*“It's just been that way from the start. […] Well, that's how it is in our family, I’d say, so nobody eats pork” (adolescent participant EKJ01, female, 16 years, IB school)*

The only male participant stated that he had no family religious practice, and another participant converted from Christianity to Islam. Several participants said that they participated in Ramadan fasting but were not sure of fasting schedules in their family. Three female participants noted that fasting at the start of Ramadan was particularly challenging, but became easier with time and familiarity.

*“At the beginning of the month it’s particularly difficult[…], but once you get back into it, it's really okay. And besides, we know what we're doing it for.” (adolescent participant EKJ01, female, 16 years, IB school)*

Religious practices were mostly unquestioned by the young people, however some participants had begun to reflect independently and expressed their own opinions, which sometimes aligned with or differed from family norms.

*“In my family, we’ve never eaten pork because of our religion. At some point, I started thinking about whether I wanted to be a Muslim. I made that decision consciously for myself.” (adolescent participant EKJ01, female, 17 years, IB school)*

Expert interviews contextualized such routines by offering that family norms and practices work as a resource for continuity and cultural identity for these adolescents within their families.

#### 3.3.5 Healthy eating and nutritional adequacy

Analysis revealed notable discrepancies between reported, observed and documented dietary behaviors, showing inconsistencies between perceived and actual nutritional practices.

Threaded through the youth interviews were viewpoints of food and nutrition that highlighted contradictions of what needs and desires the young people had surrounding the food they consumed. Some participants stated the desire to eat healthier meals in the home at the same time that they expressed a desire to consume more ready-made foods or takeout. These narratives underscored simultaneous desires or consumption that could be inconsistent. This was also seen in reporting from the triangulated data. For instance, one participant reported in their food survey that they consumed very few sweetened drinks, whereas photos and observations pointed to a more frequent consumption. In the interview with this participant, she mentioned that she usually begins her day with a glass of sweetened iced tea or water and drinks another glass of juice later on. However, over the course of the interview, she also reflected that if she were to change something about her diet, it might be to reduce her juice intake. She emphasized here that drinking more water instead could be healthier for her body and skin. These contradictions in this specific case seemed to signal limited awareness of some aspects of nutritional intake and at the same time may also be part of a developing autonomy.

The seesaw of exposure and autonomy was described in an interview with one youth center expert who described witnessing the ability to inspire and influence young people when teaching them to cook and prepare healthy recipes using food that would otherwise be thrown away. Nonetheless, this youth center coordinator indicated that they observed that when the adolescents brought their own food to prepare at the center, they resorted to easy and readily prepared options such as meat and cheese sandwiches made with a sandwich maker or frozen pizza.

Overall in the questionnaires, the young people generally rated their health positively, with reported values ranging from very good to average. Parental ratings of their children’s health, available from two parents, were also predominantly good to very good, exceeding slightly their child’s assessments.

In interviews young people expressed their everyday awareness, and in some cases vented frustration about their financial situation and ability to buy and consume certain foods. This was also witnessed during the two shopping trip observations.

Youth experts articulated a connection between financial shortage that could lead to insufficient caloric intake or less nutritious food consumption on the part of young people. One expert highlighted the limitations of influencing what was consumed in the given circumstances.

*“There was definitely some healthy food, but of course there were also things like wraps with Nutella—which obviously isn’t healthy. But the teachers do make an effort to address these things with the younger kids. With the older ones, though, you really don’t have any influence anymore.” (youth expert 1, female, between 40–50 years, social worker neighborhood youth center)*

Dietary protocols from two participants (adolescent participant EKJ01, adolescent participant EKJ06) based on 3-day food diaries capturing home-based meals only, provide a partial view of overall dietary patterns.

Within this scope, both cases show a relatively low energy intake (1,050 kcal/day and 1,610 kcal vs. 2,000 kcal/day recommended). A substantial proportion of daily energy would need to be obtained from meals consumed outside the home. Macronutrient distribution was mainly within recommended ranges, except for an elevated protein-intake in one case (adolescent participant EKJ06) (2.1 g/kg/day vs. 0.9 g/kg/day recommended). Across both cases, a similar pattern of micronutrient gaps was observed (vitamin D, E, C, calcium, potassium and iodine).

As detailed in the demographic section (section 3.2 and Figure 4), the reported BMI-SDS values showed both lower and higher weight status, indicating variability in the sample. This may be viewed alongside the diversity in dietary patterns described. At the same time, ascribed value to home cooking practices using fresh ingredients, whereas food prepared outside the home was reported in these two cases to be rarely or never consumed.

**Figure 5:**
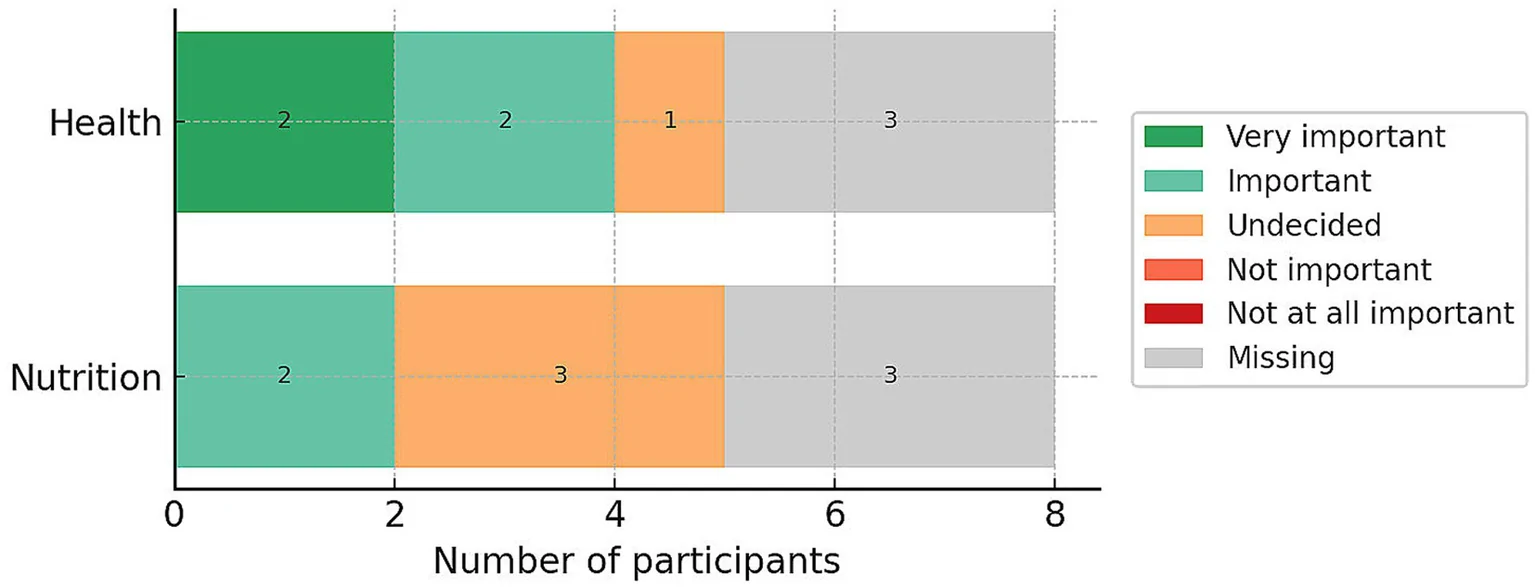
Participant self-rated importance of health and nutrition. Distribution of responses to two questions on perceived f among adolescents with nutritional deprivation (*n* = 8). Response options ranged from 1 = very important to 5 = not at all important. Bars show the number of participants selecting each category; three participants (EKJ03, EKJ07, and EKJ08) did not respond.

**Figure 6:**
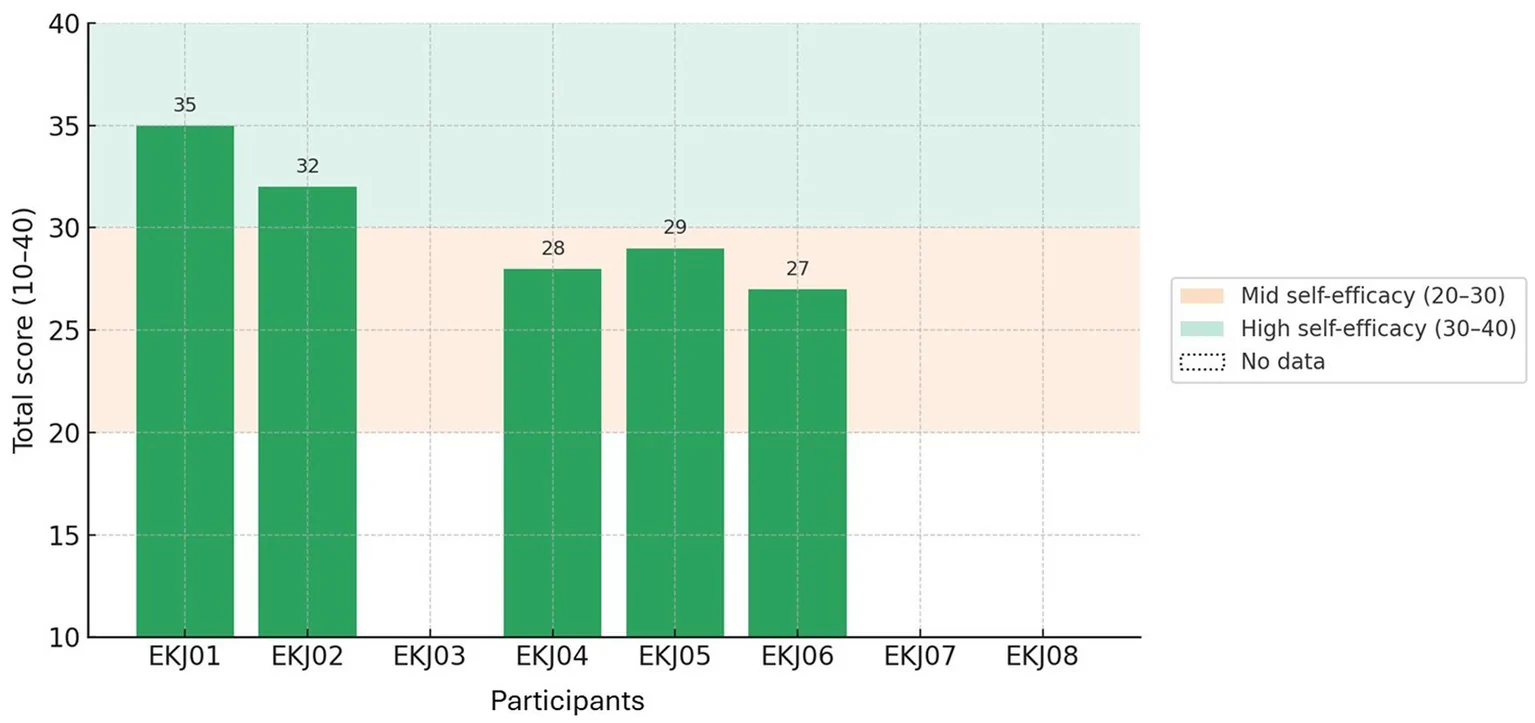
Total scores of the general self-efficacy scale (Jerusalem & Schwarzer, 10 items, range 10–40) for all participating adolescents (*n* = 8): Bars represent individual participants. Colored bands indicate the mid self-efficacy range (17–27) and high self-efficacy range (27–37). Three participants (EKJ03, EKJ07, EKJ08) did not provide data and are displayed as missing values.

Most adolescents rated their health in the self-reported questionnaire data as important, while nutrition was judged less uniformly important (Figure 5). Additionally, average general self-efficacy scores were mid-to-high (Figure 6).

### 3.4 Food in the school setting

#### 3.4.1 Availability and quality of school food

In contrast to home meals, the interviewed young people had strong opinions about the food they consumed outside of the home. In the school setting, they underscored the lack of nutritious and affordable options. Clear needs were expressed for more affordable and tasty lunch options in and around schools. In our sample of cases, most of the interviewed adolescents described eating little or nothing during school hours.

Descriptions emphasized that easily available options within or outside of the school premises, such as the school kiosk, offered only snacks and sweets for the most part. This was reflected in the photos that were taken and shared by the young people.

*“There is a cafeteria, but they only serve waffles or sweet buns or things like that.” (adolescent participant EKJ04, female, 16 years, IB school)*

Hot school lunches were not purchased by any of the participants interviewed. All cases perceived these meals to be of low quality, lacking in options and without taking cultural differences into account.

*“I’d assume that most people don't like it. […] Maybe a bit more effort could be made to offer a variety of food [instead of] always serving the same thing.” (adolescent participant EKJ04, female, 16 years, IB school)*

For those who took food with them from home, this was stated to be either prepared by their mothers or themselves in the morning or night before. Some cases mentioned buying a baked good on the way to school. One participant stated that she does not take food to school with her specifically if it is a short school day.

*“So during my long lunch break, I either go home and eat my mom's cooking, or I go out with friends for a quick bite and have a small pizza or something.” (adolescent participant EKJ04, female, 16 years, IB school)*

One exception to this was a participant case that attended a trade school where the pupils prepare breakfast and lunch themselves as part of the educational model. This was described to create a connection between students, money and greater knowledge about groceries while shopping together as part of this school environment.

*“I learned that at school because cooking meant we had to do the shopping ourselves.” (adolescent participant EKJ07, male, 17 years, vocational school)*

#### 3.4.2 Relevance to nutritional adequacy

Daytime food intake at school was often reported in interviews to be limited or skipped, often consisting of small snacks rather than full meals. At the same time, the food diaries focused on home-based intake representing both the portion of intake adolescents reliably consumed. However, this also served as an incomplete picture of total food consumption. Here, the interview accounts underscore the finding that school-day intake tends to focus on snacks. Taken together with the limited snapshot of food consumption reported in the diaries, this suggests that adolescents’ overall daily intake may be shaped predominantly by home meals (see “Nutritional adequacy” section), as school was not reported to consistently provide substantial or nutritious food.

### 3.5 Eating with peers

#### 3.5.1 Influence from friends

Eating with friends elicited animated descriptions from the young people. Snack trends shared between friends, such as chips or cinnamon buns, enabled the young people to explore new places and try new flavors together. However, these snack trends were seen by our cases as expensive and not always accessible on the available budget. To circumvent this challenge, the young people talked about strategies they developed such as taking turns paying and or sharing resources to make it affordable for everyone.

*“Yes, but [Takis] are expensive. So, we just shared them.” (adolescent participant EKJ02, female, 16 years, IB school)*

Despite these strategies, some participants cited feeling peer pressure or the desire to consume more foods outside of the home, but felt limited ability to do so.

#### 3.5.2 Eating during free time, hobbies and activities

Asked about the influence of hobbies, sports or activities on eating habits, most participants expressed that these had little influence on what they consumed. Our sample cases of young people generally did not mention partaking in many extracurricular activities, but rather mentioned that they do not have much free time. Time spent with friends was described to take place at or just after school, whereas evening time is spent with family, or used for school work.

*“Well, I only hang out with my friends at school and after school. But I don't go out with girlfriends to eat or anything like that.” (adolescent participant EKJ05, female, 15 years, secondary school)*

*“Actually, I don't meet up with my friends that often because I have strict parents.” (adolescent participant EKJ01, female, 16 years, IB school)*

Parental rules or restrictions were cited as one reason for the evening home routine.

One notable exception was given by a participant who attended a fitness studio. In this case, she was introduced to fitness and protein foods, such as protein bars and shakes, by an older brother who had moved outside the home. This participant conceded that her fitness routine and associated foods were amenable to the plans and demands of her family, as well as the financial support and provision of goods by her brother.

#### 3.5.3 Social media and food trends

During all interviews, the topic of social media and the influence on food consumption was asked. With the exception of one participant, all cases said that social media, especially the platform TikTok, plays a major role in their perception of nutrition and trendy foods. Social media was exclusively consumed in these descriptions among peers and there was little interaction or role that social media played within the family food dynamics. This was bolstered by quantitative findings in the self-reported questionnaires which align with cases’ account of importance of own health as well as their confidence in making food choices with friends (see Figure 4 on the self-reported health importance and Figure 5 on self-efficacy). Interviews and observation cases provided additional context on how this is done within available resources.

### 3.6 Financial awareness and resource limitations

#### 3.6.1 Awareness of limited resources

All cases showed clear awareness of their family’s financial limitations. They mentioned rising food prices, the need to compare offers, and buying items only when affordable. The young people described making decisions in everyday food choices based on what the family could reasonably buy. In some cases, participants explained that they refrained from expressing personal preferences to avoid additional cost or conflict about cost.

Corresponding behavior was observed during the two accompanied shopping trips. Personal preferences of the interviewed young people were mentioned to sometimes be taken into consideration for family meals, but these decisions were reported to be balanced against cost, preferences of other family members and what was deemed as an acceptable family meal. One participant actively stated his frustration with the rising food prices that he connected with having an impact on his financial situation.

*“A lot of what we buy are sale items. Because, to be honest, everything is ridiculously expensive everywhere, and that's just annoying because at some point you just don't feel like buying anything anymore.” (adolescent participant EKJ07, male, 17 years, vocational school)*

#### 3.6.2 Family budgeting

The two observations of shopping showed situations where purchases were carefully planned, involving choosing different stores to find the best offers, something that was underscored in interviews. In the parental questionnaire, both participating families rated their financial management positively, with high finance index scores (4/5 and 5/5).

## 4 Discussion

Our interviews with six young people from low-income households highlighted the perspectives of young people on their health and nutrition when confronted with resource constraints. Two families gave insights into meals and grocery shopping, while additional viewpoints and context to described experience was augmented by expert interviews with youth workers. Added exploratory and descriptive accounts of reported health, nutrition and financial data helped provide a fuller picture to the reported experiential accounts. Young people’s perspectives with added contextualization of those around them is missing from the literature; especially how young people themselves view their health and nutrition. These accounts describe how family meals in the home provide a regular meal routine, have a parent-determined structure that in our sample was described as linked to cultural identity or religious practices. Outside the home, after school and with friends, food preferences highlighted snacks and burgeoning autonomy about food consumption. These situations were described as ways to bond with peers over trends and trying new tastes. While our case sample ascribed health as important to them, the importance of nutrition was not as clearly or specifically described. The interconnection between health and socioeconomic conditions becomes clearer when viewed in its totality. Our young participants were clear-eyed about their lack of financial resources, leading both to perceived restrictions about spending and strategies to cope and fit in with more affluent peers.

The family setting was revealed to be the most important food environment of the interviewed adolescent cases. Regular home cooking with fresh ingredients was described, and the cooking parent, in all cases the mother, played the central food-the central role as food arbiter role. Research shows that frequent family meals and meals prepared at home are associated with better dietary intake in children and adolescents independently of SES (54–56). In an umbrella review, a higher family meal frequency additionally improved obesity, mental health and risk behavior as well as academic performance of youth (57). In our sample, cultural traditions and religious practices were underscored in the home meals described, a finding in line with evidence that culture and religion influence diet and food preferences in families (58, 59). Almost all adolescents adapted to established household norms, reflecting findings in the literature that the parents remain key food gatekeepers of family food environments that have been seen to direct adolescents’ eating practices (57, 60).

In contrast, the school food environment was perceived as limited and of low quality across all of our young participant cases, resulting in most participants disclosing that they avoided eating meals at school unless this was brought from home. This is consistent with studies reporting low acceptance of school meals among adolescents due to perceived poor taste, limited variety and dissatisfaction with quality (61, 62). Another complaint was a lack of availability of foods to match tastes, also reported in previous research (63). The reliance on small snacks or meal skipping because of lacking or undesired school setting options is an area of needed further research, especially in the context of lower SES. If less food is being consumed during school hours, this could be an area of risk for nutritional inadequacy. One systematic review found that up to 83% of young adults reported skipping any meal during the day depending on setting (64). Several studies have also shown that skipping breakfast was associated with lower nutritional adequacy in children and adolescents (65–67).

Home-based dietary intake was completed and recorded for two adolescents in this study. In both cases, the total energy consumed at home did not reach DGE-recommendation; making meals outside the home, e.g., school meals necessary for nutritional adequacy in these two adolescents. This underlines the potential importance of nutritionally adequate and acceptable school meals that adolescents also signaled demand for in the interviews. International studies have also shown that school meal programs are associated with higher diet quality and improved nutrient intake in children and adolescents (68, 69), thereby demonstrating how policies for meal provision may provide long term returns for the health of young people (70). In addition to improved nutrition, breakfast and school meals have been shown to impact cognition and academic achievement as well as attendance (71, 72).

Our sample described wanting to eat and try new foods with peers but they felt constrained by budget limitations, in line with international literature on snacking behavior in youth (73). Social eating occurred less frequently than preferred by participants, and trend foods typically consisted of snacks, consistent with evidence elsewhere that peers often promote consumption of energy-dense, nutrient-poor foods and that snack foods are a central part of adolescent social eating (74). However, our insights give only a small window into the topic of snacking. More data is needed to understand how snacking practices differ in different populations and how this may impact nutritional status compared to wealthier peers.

The topic of financial awareness was central across all the participants we interviewed. The adolescents demonstrated a clear understanding of constrained budgeting conditions. One strategy our cases employed was to adjust their own wishes according to availability of different foods, aligning with a study showing that adolescents in low-income households often adapt to limited resources (75). How this may affect adolescents in the long term is an area needing greater exploration to better understand the impact on long-term resiliency, resourcefulness and/or burdens of their nutritional wellbeing.

Our findings align with evidence from European and high-income settings, showing that youth from low SES households make food choices within environments constrained by cost, availability and social context, which in turn shape dietary patterns and health inequalities (76–78). In our cases, the reduced acceptance of school food, taken together with a tendency toward skipping meals and snacking during the day, displays patterns described in previous research, where youth participation in school meals is influenced by food preferences, social context, and availability, and where snacking has been connected to the omission of meals (79, 80). Likewise, the awareness of financial limitations and the development of coping strategies align with research showing that adolescents adapt their eating practices to limited resources (81). Our study cases add individual perspectives on how these conditions can be experienced and dealt with in everyday life.

Together, our results suggest that for our cases, the adolescents’ nutritional wellbeing was strongly influenced by the intersection of structural limitations and family meal provision. Others have shown that adolescent food insecurity and diet quality are shaped by different levels of environmental factors (78). While the families in this study provided regular home meals, nutritional gaps outside the home were implied, particularly at school, which aligns with research seen elsewhere (82, 83). Focusing on structural improvements to school food environments and community settings where adolescents exercise increasing autonomy over food choices is an important area of research highlighted by this exploration and underscored by others (84, 85).

### 4.1 Tensions between data sources

Of the two complete reports given by parents, daily home-cooking with fresh foods aligned with what the adolescents themselves reported along with the observational data. However, a more critical viewpoint emerged from the expert interviews. These highlighted the pressures the experts perceived that families faced given their low SES. Careful price comparison and searching for special offers in multiple stores were also witnessed in the two shopping observation sessions. In some cases, instances of self-reported accounts did not fully align and exhibited paradoxes in what was reported. For instance, most of the adolescents interviewed stated that their health and healthy eating were important to them. At the same time, photographed sweetened beverages such as sweetened ice tea were presented as healthy options. Discrepancies also appeared between positive health self-ratings and objective risk markers. Adolescents and their parents mostly rated own/child’s health as good or very good, while BMI ranged from underweight to obesity. Misreporting of energy intake is a known phenomenon in public health (86, 87). How such misreporting may be differentiated or interpreted for adolescents from families with fewer resources is an area of potentially rich exploration especially in understanding whether certain tendencies for underreporting or overreporting are evident. Using multiple perspectives in the data collection may have benefits of understanding this phenomenon more comprehensively, but also needs greater scrutiny.

### 4.2 Limitations of the study

This study comes with several limitations to be acknowledged when interpreting the results. The difficulties encountered during the recruitment process situate the findings within broader conditions affecting youth participation in health initiatives. Our recruitment required substantial engagement through different youth institutions, confirming that standard outreach approaches may not reach the target population. This is reflected in scientific literature showing that successful engagement in low-income settings demands partnering with local intermediates trusted by low-income populations (88, 89). Time constraints, competing priorities, and potential concerns about stigma have been found to limit participation in preventive programs and research, even when interest is present, in line with reviews showing that practical burdens (time, transport, family responsibilities). Distrust or fear of stigma are common barriers to participation in health research among socially disadvantaged and vulnerable populations (90, 91).

Our case sample was small and exploratory and did not reach the intended number of participants. This limited our ability to capture the full heterogeneity of experiences of adolescents in low-income families. Additionally, the participants that took part did not provide all data, resulting in incomplete cases across study outcomes (e. g. such as food diary of two adolescents). To reach households with low SES, inclusion criteria required families to receive government financial support, excluding families whose combined income put them above the threshold for institutional subsidies eligibility. Although it is estimated that subsidies bring those in the lowest income bracket adequate support for families to afford and maintain an adequate living standard, families with incomes slightly above the threshold allowed for financial assistance may be at a greater disadvantage yet were ineligible for our study. Families agreeing to participate may also represent a more organized and engaged subgroup, subsequently leaving less connected families underrepresented. The sample demographics of our cases differed from low-income household distribution in Germany more broadly with all but one case coming from an immigrant background. Berlin has a vastly larger immigrant population compared to other German states that are at greater risk for wealth inequities (92), which may in part explain the diversity of our sample. This could have specific consequences on the experiences of what, when and how food is consumed. Moreover, the largest growing segment of *Bürgergeld* (now called *Grundsicherungsgeld,* Basic Income Support) subsidy-recipients are those with immigrant backgrounds, yet our sample does not reflect the current makeup in Germany (15). Our cases had a high proportion of adolescents attending higher level secondary schools (IB schools, Gymnasiums) that contrasts with education level in populations with lower SES. Across Germany, only 26.7% of children from disadvantaged backgrounds attend higher secondary school (93), which contrasts our cases, where 4 of the 6 adolescents are attending such schools. Our strategic recruitment yielded only recruit one male participant. Additionally, this study relied on self-reported data, including dietary recall and cooking and financial practices. Such data are subject to recall bias and social desirability effects, which may underestimate problematic behaviors. Finally, the response categories for the frequency of home cooking, ready-meal cooking and outside meals are a limitation; while suitable for home-cooked meals, the gap between “less than three times a week” and “almost never” lacks the granularity to distinguish between rarely and never dining out. Overall, our cases gives an in-depth snapshot of various dietary practices, but lack comprehensive breadth and theoretically-saturated experience.

Despite these limitations, the integrated approach linking qualitative accounts with quantitative nutritional indicators and contextual expert perspectives provides valuable and underrepresented insights into the food environments of adolescents facing financial hardship.

The cases in this study with young people from low-income households in Berlin provided an in-depth snapshot of their everyday experiences with food, health and nutrition. Well-aware of their financial limitations, these youths adhered to individual family eating norms, while they developed strategies for coping with disadvantages compared to wealthier peers. The school environment and social media were hindrances to healthier nutrition while meals at home was the primary source of meal consumption. Lack of access to healthy, affordable food in and during school hours highlight a particular discrepancy between low income pupils and their wealthy peers.

## Data Availability

The raw quantitative data supporting the conclusions of this article will be made available by the authors upon individual request. Requests to access the data should be directed to the corresponding author. Qualitative data will not be shared in order to protect participant identity.

## Footnotes

1 German subsidies have had multiple conceptual and terminology changes over the last two decades, supported by a legal framework embedded in Germany’s constitution that guarantees the right to a decent subsistence level. Originally this benefit was named unemployment benefit II (*Arbeitslosengeld II*) and known informally as *Hartz IV*, named after the federal-state body (the Hartz Commission) that analyzed the need for reform and developed concrete proposals that were enacted into law as of 2005. In 2023 this unemployment entitlement law was enacted and called the Citizen’s Benefit Act (*Bürgergeld-Gesetz*), which aimed to provide a basic income support. This was slightly restructured and renamed as Basic Income Support (*Grundsicherungsgeld*) as of July 1, 2026 (94–96).
