# Supplementary material for: Perspectives of adolescents from low-income households in Berlin—nutrition described by food affordability, household routines and autonomy: results from an exploratory qualitatively-driven mixed-methods study of cases
Source: Front Public Health. 2026 Aug 28;14:1791106. doi: 10.3389/fpubh.2026.1791106 (PMC13562026; doi:10.3389/fpubh.2026.1791106)
Supplement: Supplementary file 1 [file Supplementary_file_1.DOCX]

Supplementary Material

Contents

[1	Supplementary – Data	2](#_Toc219710434)

[1.1	E-Kids Study protocol	2](#_Toc219710435)

[1.2	E-Kids incentive scale	13](#_Toc219710436)

[1.3	E-Kids youth interview guideline	14](#_Toc219710437)

[1.4	E-Kids expert interview guideline	16](#_Toc219710438)

[1.5	E-kids participatory fieldnote form	17](#_Toc219710439)

[1.6	E-Kids coding tree	21](#_Toc219710440)

[2	Supplementary – Figures	24](#_Toc219710441)

[2.1	E-kids – Photovoice	24](#_Toc219710442)

# 1 Supplementary – Data

## 1.1 E-Kids Study protocol

**Study protocol for the E-Kids project**

**Explorative assessment of the nutritional situation**

**of children and young people**

**in families affected by poverty in Berlin**

Prof. Dr. med. Georg Seifert, Dr. rer. medic. Wiebke Stritter, Dr. med. Gonza Ngoumou

Clinic for pediatrics with a focus on oncology/hematology/SZT

Charité University Medicine Berlin

**Contents**

[**Background**	3](#_Toc134543962)

[**Poverty, health and nutrition**	3](#_Toc134543963)

[**Food poverty**	4](#_Toc134543964)

[**Status quo of the poverty situation**	5](#_Toc134543965)

[**Studies on food poverty**	6](#_Toc134543966)

[**Questions and objectives**	7](#_Toc134543967)

[**Methodology of data collection and analysis**	7](#_Toc134543968)

[**Design**	7](#_Toc134543969)

[**Participants**	7](#_Toc134543970)

[**Data collection**	8](#_Toc134543971)

[**Data evaluation**	8](#_Toc134543972)

[**Data protection**	9](#_Toc134543973)

[**Data management/access control**	9](#_Toc134543974)

[**Financing and economic interests**	9](#_Toc134543975)

[**Publication**	9](#_Toc134543976)

[**References**	10](#_Toc134543977)

**Background**

It is well known that the issues of poverty, health and nutrition are interrelated. Essentially, it is assumed that poverty leads to restrictions in health and health behavior as well as in nutritional behavior and nutritional quality. This topic is particularly relevant for children and adolescents, also due to the great preventative importance of a healthy diet. The consequences of poverty have a direct impact on the morbidity and development of children and young people. In Berlin, with its very heterogeneous social structure, the topic is also of great importance.

The data landscape on health, nutrition and poverty is diverse. Connections between the areas have been proven, but the overall picture remains fragmented. Little research has been carried out into the detailed recording of backgrounds, nutrition and lifestyles, as well as the individual causes, consequences, problems and solutions of affected families. The situation is similar with analyses of the experiences of the children and young people themselves.

The aim of this project is to examine the living environment of these families and, in particular, of children and young people. In addition to the quantitative recording of nutrition and objective and subjective health, the qualitative investigation of experiences, difficulties, resources and proposed solutions for families are of particular interest. The aim is to show the complexity of food poverty in Berlin and to look for solutions together with those affected.

**Poverty, health and nutrition**

Poverty and health are closely linked. The negative effects of poverty on health are already evident in childhood, particularly in terms of mental health, developmental delays, obesity and dental health. Even if the extent of health impairments in childhood, the healthiest of all phases of life, is still low, there are already clear differences in the assessment according to social situation. It is important for the further course of development that the health problems associated with poverty are usually chronic and are associated with risks for development, but also for health into adulthood. In terms of health-related behavior, poor children and adolescents are more frequently exposed to risk factors from the outset. These include tobacco consumption, an unhealthy diet, low levels of physical activity, but also a lower uptake of preventive check-ups. Longitudinal studies show that the accumulation of health problems and risk factors often only manifest themselves in young adulthood (Laubstein & Sthamer, 2020)

A survey of the health data of children between the ages of 5 and 6 when they start school in Germany shows that children from socially disadvantaged families are more likely to have physical, cognitive, emotional, language and motor impairments than children from families without social need (Groos et al., 2020){Groos T, 2020 #2}. A similar conclusion was reached by the evaluation of the results of the
school enrolment examinations in Friedrichshain-Kreuzberg Berlin for the years 2010 to 2019 came to a similar conclusion (Beil, 2021). Lampert et al. (Lampert & Kuntz, 2019) examined the effects of poverty on the health status and health behavior of children and adolescents in the second follow-up survey of the Study on the Health of Children and Adolescents in Germany (KiGGS wave 2, 2014-2017). This revealed that children and adolescents from families affected by poverty are more frequently affected by health restrictions. In particular, both subjective and objective permanent health restrictions were more common. At the same time, it was found that children and adolescents from low-income families suffered more frequently from mental health problems than children and adolescents from middle- and higher-income households. In addition, clear differences in eating habits were found. Children and adolescents from low-income families consumed less fruit and vegetables and more sugary drinks. They were also less physically active and more likely to be overweight (Lampert & Kuntz, 2019).

Data from the USA has established a clear link between poverty and food insecurity (Bhattacharya et al., 2004; Hanson & Connor, 2014). On the one hand, this is attributed to the prevalence of "food deserts" in low-income neighborhoods in the USA. On the other hand, low-income families have less money available for food. At the same time, it has been shown that calorie-dense, nutrient-poor foods have a lower cost per calorie than nutrient-rich foods such as fresh fruit and vegetables (Drewnowski, 2010). In addition, low-income households often have limited financial management skills (Gundersen & Garasky, 2012). Another survey of young people from the USA found that young people from food insecure households were more likely to find that healthy food was inconvenient and did not taste good than young people from food secure households (Widome et al., 2009).

**Nutritional poverty**

Food poverty affects various dimensions of everyday life. A distinction can be made between material and social food poverty. Material food poverty means that there are insufficient financial resources to provide adequate food and a balanced diet. Social food poverty includes the possibility of social participation in the form of an invitation to a birthday party, a joint visit to a restaurant or eating out, but also the possibility of receiving a warm lunch from the 7th grade onwards. Feichtinger (1995) further subdivided the dimensions of food poverty and named them as follows.

| Dimensions | Functions |
| --- | --- |
| physiological | Supply of energy and nutrients |
| social | Social organization, integration and demarcation, social security, communication |
| cultural | normative value systems, eating habits and customs, edibility, taste |
| psychic | Pleasure, emotional security, compensation, self-esteem |
| economical* | Income available for nutrition as an economic and socio-cultural access entitlement |
| temporal* | Effects of frequency and duration of certain nutritional situations over time |

Table 1: Dimensions and functions of nutrition (according to Feichtinger 1995: 292). The economic and temporal dimensions, which are marked with an asterisk in this table, are not themselves functions of nutrition, but have a significant influence on it (cf. ibid.).

What was left out of Feichtinger's dimensions is the issue of education. Both knowledge (product knowledge - e.g. food storage, preservation, utilization of leftovers, nutrients) and skills (cooking skills) can play a relevant role in relation to food poverty. It can be assumed that there is a connection between a low income or a risk of poverty and a low level of education (Barlösius et al., 1995).

An important point that plays a role in food poverty is the distribution of expenditure across different areas of life. Relative to income, families at risk of poverty spend a large proportion of their income on food, but significantly less in absolute terms than families who are not at risk of poverty (Aust & Andreas, 2020).

**Status quo of the poverty situation**

According to the Federal Statistical Office (DESTATIS), around 13.0 million people in Germany were at risk of poverty in 2021. This corresponds to 15.8% of the German population (DESTATIS, 2022).

The concepts of relative income poverty and the concept of SGB II benefit receipt are often used to describe poverty. However, due to the limited number of cases, it is hardly possible to differentiate according to other characteristics such as age or family type at district level (Laubstein & Sthamer, 2020).

Berlin has long had significantly higher poverty rates than Germany as a whole. Families of single parents and families with several children are particularly often affected by poverty. Families with several children are at a comparatively high risk of poverty in Berlin. In addition, discrepancies between the rates of income poverty and SGB II receipt indicate a high proportion of hidden poverty in this group. What is specific to Berlin is that the incidence of poverty does not decrease as children get older, but is high across all age groups (Laubstein & Sthamer, 2020).

More than one in five children in Germany is growing up in poverty. That is 2.8 million children and young people under the age of 18. Child poverty in Germany has been an unsolved structural problem for years (Chemnitz et al., 2021). There are hardly any differences between the various age groups. Young adults are particularly likely to live in relative income poverty (Laubstein & Sthamer, 2020).

In Berlin, child poverty is unevenly distributed, both at district level and at the level of planning areas within the districts. Although the research findings on the effects of child poverty in Germany are extensive, there are still data gaps. These relate to the stringent pursuit of a life course perspective for institutional transitions for all age groups as well as an analysis of the target group with its intervention and prevention needs (Laubstein & Sthamer, 2020). The results from the German Socio-Economic Panel Study (SOEP), which has recorded around 15,000 households since 1984, provide a broader picture of poverty in families. In addition to long-term social trends, this also allows group-specific developments in life courses to be analyzed particularly well (DIW-Berlin). The insights gained from this, combined with data from other sources, could help to learn more about the poverty situation in families.

**Studies on food poverty**

To date, there have only been a few systematic surveys on food poverty in families in Germany. Often, only individual parameters that contribute to food poverty are recorded as a partial survey or the influence of poverty on health as a whole is examined, such as in the second wave of the KIGGS study (Lampert & Kuntz, 2019).

A report by the Heinrich Böll Foundation refers to studies that show that poor households have a much smaller variety of food and that cheap, filling foods are preferred to fruit and vegetables. A total of 11% of German households in the lowest income groups state that they cannot afford a full meal every other day (Chemnitz et al., 2021).

A qualitative study on the assessment of poverty in families (GESA) (Lehmkühler & Stephanie, 2002) from 2002 examined 15 poor households with regard to their eating habits in the Giessen hotspot. The results clearly show how heterogeneous the group of low-income households is and depict different manifestations of poverty in the families. Many of the families surveyed were affected by educational and training poverty. In the poor households surveyed, food played a rather subordinate role, as the family members were primarily preoccupied with other problems such as childcare, being alone or family conflicts. The results point to financial bottlenecks, in which the income is often no longer sufficient for a healthy diet at the end of the month. The diet becomes very one-sided during the so-called "drawing or stretching weeks". Some of those affected report hunger (Lehmkühler & Stephanie, 2002).

The author reports that meals in the families are generally less varied and diverse and also contain a low proportion of whole grains. In addition, the families studied showed an increased consumption of sugar, salt and fat. At the same time, food was often eaten in a hurry and had a different significance than in families not at risk of poverty. Lehmkühler et al. quote: "The results of the study show a dietary behavior that consists more of pure necessity to satisfy the feeling of satiety as well as of haste and isolation than of visual pleasure, enjoyment and fun in eating and drinking." (Lehmkühler & Stephanie, 2002).

Previous studies on food insecurity in children have rarely recorded how the children and young people themselves perceive their experiences. A study from London used a mixed methods approach to investigate the experiences of children from food insecure households. The children were clearly aware of the problems despite their parents' attempts to shield them. The associations of low-nutrient but inexpensive takeaway food emerged as a notable consequence of food insecurity (Harvey, 2016). A 2020 study revealed several dimensions of psychological distress in children living in food insecure households, including worry, anger, frustration, shame, family isolation and sadness (Leung et al., 2020).

It is clear that different methodological approaches need to be used in an interdisciplinary way in order to obtain a differentiated picture of poverty in families. This is where this project comes in and aims to use a mixed methods approach to gain insights into the needs of families at risk of poverty and possible interventions.

**Questions and objectives**

Through an explorative survey and description of the nutritional situation of children and adolescents in families affected by poverty in Berlin, a comprehensive understanding of their situation with regard to nutrition and health is to be achieved. The following questions are to be answered as part of a qualitative study of the experiences of children and young people and their families, as well as the difficulties and resources in relation to nutrition and eating:

- How do children and young people affected by poverty and their families describe their living situation with regard to nutrition (and the various dimensions of food poverty)?
- How do you assess the state of health (physical and mental) of your children?
- What kind and extent of support do the families want and need?

Qualitative data is primarily collected in the form of interviews, participant observation and photo documentation of food consumed.

The qualitative data are analyzed by means of a deductive-inductive thematic analysis (Braun & Clarke, 2006) analyzed.

In addition, quantitative data in the form of questionnaires on subjective health status, information on dietary habits, attitudes and self-efficacy with regard to nutrition and the use of scarce resources are collected and analyzed according to descriptive criteria.

**Methodology of data collection and analysis**

**Design**

The project is based on an explorative mixed-methods design with a focus on qualitative data collection and analysis in order to adequately address the complex, multi-layered nature of the topic of nutrition in the context of poverty. This methodological approach allows a differentiated picture of the nutritional situation of families at risk of poverty in Berlin to be shown, and needs for support and interventions from the families' perspective to be recorded.

**Participants**

Inclusion criteria are as follows:

- Young people aged 14-17 with a declaration of consent from parents/guardians at least one parent (legal guardian) is a recipient of ALG 2/Hartz IV
- Sufficient knowledge of German
- Willingness to participate in at least one interview, photo documentation of the meal and completion of questionnaires (data collection stage 1)

Exclusion criteria for participation in the study are

- existing diseases and health conditions that could impair participation in the study
- Participation in other studies, insofar as they could hinder participation in the study

A total of approx. 10-15 families are to be included.

**Data collection**

- Data collection takes place in two stages. Only families who agree to at least the first stage of data collection are included.

**Stage 1**

- Documentation of the food consumed by taking photos of the adolescents for 1 to 3 days (see Photovoice, (Strack et al., 2004)). The aim here is to record what the young people eat independently. Both meals at home and meals in the school canteen or snacks should be documented. The aim is to gain an overview of eating from the young people's perspective and to sensitize the young people to food and food intake in order to talk to them about food, eating situations and food intake in the subsequent interviews, in line with the Photovoice methodology.
- Qualitative problem-centered interviews (Witzel, 2000) primarily with the young people. The interview can take place with or without parents, depending on the preference of all participants.
- Quantitative questionnaires on the subjective health situation of young people in self-assessment by the young people;
- Young people's attitude towards nutrition (mindset);
- Self-efficacy in relation to nutrition by young people;
- Nutrition questionnaire by the young people;
- Demographic data from young people and parents/caregivers;
- Anthropometric data from adolescents and parents/caregivers.

**Stage 2**

- Nutrition protocol with the person in charge of the household;
- Focused participant observation of a shopping and/or eating situation in the family;
- Quantitative questionnaires on the subjective health situation of adolescents as assessed by parents/caregivers;
- Financial management skills of the person in charge of the budget.

**Data evaluation**

The interviews are transcribed and evaluated together with the food diaries, observation and shopping logs as well as anamnesis and information from the partially standardized questionnaires using thematic analysis (Braun & Clarke, 2006).

The food selection and nutrient supply is determined from the shopping or food diaries and supplemented by the information in the interview and the shopping documentation. In addition to nutritional and shopping behavior, this can also provide information about shared meals, social interaction and participation. During the evaluation, the results are assigned to the various dimensions of food poverty and combined with the identified needs for interventions in individual families and their characteristics.

**Data protection**

All data is stored and locked on a protected (audio) data carrier. Only people from the research team have access.

The audio files are transcribed under a pseudonym.

The pseudonym will be preserved in the further course of the study. The pseudonymization table is only accessible to the study team. Publication of the data in specialist publications also takes place exclusively under pseudonyms.

**Data management/access control**

The data is stored on a protected server of the research-managing institution and is only accessible to project staff. The department's computers are each set up with a personal password and a screen saver with password entry on reactivation. This prevents potential access to data by third parties in the event of a staff member's brief absence. Written data is stored in lockable cabinets. Only the project manager and project staff will have access to this data.

**Financing and economic interests**

The study is financed by third-party funds from the Senate Department for the Environment, Mobility, Consumer and Climate Protection, funding area Implementation of the Berlin Food Strategy.

**Publication**

The study will be published in recognized journals, irrespective of the results.

**References**

Aust, & Andreas. (2020). *[Poor, dependent, marginalised. A study into the hardships of living on Hartz IV benefits]*. Deutscher Paritätischer Wohlfahrtsverband – Gesamtverband e. V. <https://www.der-paritaetische.de/fileadmin/user_upload/Publikationen/doc/expertise-armutsfest-bedarfsgerecht_2020_web.pdf>

Barlösius, Feichtinger, & Köhler. (1995). *[Nutrition in poverty. Health, social and cultural consequences in the Federal Republic of Germany]* (Edition Sigma Berlin, Ed.).

Beil. (2021). *[Results of the pre-school health checks in Friedrichshain-Kreuzberg for the years 2010–2019]*. Bezirksamt Friedrichshain-Kreuzberg Berlin. Retrieved 27.05.2026 from <https://www.berlin.de/ba-friedrichshain-kreuzberg/politik-und-verwaltung/service-und-organisationseinheiten/bezirkliche-planung-und-koordinierung/planungs-und-koordinierungsstelle-gesundheit/arbeitsgebiete/artikel.166273.php>

Bhattacharya, J., Currie, J., & Haider, S. (2004). Poverty, food insecurity, and nutritional outcomes in children and adults. *J Health Econ*, *23*(4), 839-862. <https://doi.org/10.1016/j.jhealeco.2003.12.008>

Braun, V., & Clarke, V. (2006). Using thematic analysis in psychology. *Qualitative Research in Psychology*, *3*(2), 77-101. <https://doi.org/10.1191/1478088706qp063oa>

Chemnitz, Dewitz, & Weigelt. (2021). *[Poverty leads to hunger. Facts on global nutrition 2021]*. Heinrich Böll Stiftung und TMG Research. <https://www.boell.de/sites/default/files/2022-01/Boell-Armut-macht-Hunger_V01_kommentierbar.pdf>

DESTATIS. (2022). *[Relative risk of poverty in Germany stands at 15.8% in 2021]*. Retrieved 27.05.2026 from <https://www.destatis.de/DE/Presse/Pressemitteilungen/2022/08/PD22_327_634.html#:~:text=327%20vom%204.,%2C8%20%25%20der%20Bev%C3%B6lkerung%20Deutschlands>

DIW-Berlin. *Sozio-oekonomisches Panel (SOEP)*. Retrieved 20.10.2022 from <https://www.diw.de/de/diw_01.c.412809.de/sozio-oekonomisches_panel__soep.html>

Drewnowski. (2010). The cost of US foods as related to their nutritive value. *Am J Clin Nutr*, *92*(5), 1181-1188. <https://doi.org/10.3945/ajcn.2010.29300>

Feichtinger, & Elfriede. (1995). [Poverty and Nutrition in an Affluent Society: The Landscape of a Problem]. In Barlösius, Feichtinger, & Köhler (Eds.), *Nutrition in poverty. Health, social and cultural consequences in the Federal Republic of Germany.* (pp. 291–305). Edition Sigma.

Groos, Trappmann, Kersting, & Jehles. (2020). *[The illusion of equal opportunities. Selected findings from local government impact research using microdata]*. Bertelsmann Stiftung. <https://www.bertelsmann-stiftung.de/fileadmin/files/BSt/Publikationen/GrauePublikationen/KeKiz_WB13_gruen_Illusion_der_Chancengleichheit_2019.pdf>

Gundersen, & Garasky. (2012). Financial management skills are associated with food insecurity in a sample of households with children in the United States. *J Nutr*, *142*(10), 1865-1870. <https://doi.org/10.3945/jn.112.162214>

Hanson, & Connor. (2014). Food insecurity and dietary quality in US adults and children: a systematic review. *Am J Clin Nutr*, *100*(2), 684-692. <https://doi.org/10.3945/ajcn.114.084525>

Harvey, K. (2016). "When I go to bed hungry and sleep, I'm not hungry": Children and parents' experiences of food insecurity. *Appetite*, *99*, 235-244. <https://doi.org/10.1016/j.appet.2016.01.004>

Lampert, T., & Kuntz, B. (2019). Auswirkungen von Armut auf den Gesundheitszustand und das Gesundheitsverhalten von Kindern und Jugendlichen. *Bundesgesundheitsblatt - Gesundheitsforschung - Gesundheitsschutz*, *62*(10), 1263-1274. <https://doi.org/10.1007/s00103-019-03009-6>

Laubstein, & Sthamer. (2020). *[Poverty among children, young people and their families in Berlin]*. Y. a. F. A. Senate Department for Education.

Lehmkühler, & Stephanie. (2002). [The Giessen Nutrition Study on the dietary habits of households living in poverty (GESA): qualitative case studies]. <https://doi.org/http://dx.doi.org/10.22029/jlupub-17057>

Leung, Stewart, Portela-Parra, Adler, Laraia, & Epel. (2020). Understanding the Psychological Distress of Food Insecurity: A Qualitative Study of Children's Experiences and Related Coping Strategies. *J Acad Nutr Diet*, *120*(3), 395-403. <https://doi.org/10.1016/j.jand.2019.10.012>

Strack, Magill, & McDonagh. (2004). Engaging Youth through Photovoice. *Health Promotion Practice*, *5*(1), 49-58. <https://doi.org/10.1177/1524839903258015>

Widome, Neumark-Sztainer, Hannan, Haines, & Story. (2009). Eating when there is not enough to eat: eating behaviors and perceptions of food among food-insecure youths. *Am J Public Health*, *99*(5), 822-828. <https://doi.org/10.2105/ajph.2008.139758>

Witzel, A. (2000). The Problem-centered Interview [inquiry; evaluation; deduction; induction; narration; dialogue; semi-structured interview; problem-centered interview]. *Forum: Qualitative Social Research*, *1*(1). <http://www.qualitative-research.net/index.php/fqs/article/view/1132>

## 1.2 E-Kids incentive scale

| Data collection | Time | Family size | Food voucher compensation amount |
| --- | --- | --- | --- |
| Stage 1: Expenses Photo documentation, interview, questionnaire | Approx. 3 hrs. | Families with 2 persons | 200 € |
|  |  | Families with 3 persons | 250 € |
|  |  | Families with 4 or more persons | 300 € |
| Stage 2: Participation in shopping and/or eating situation, food log | 3 – 6 hours | Families with 2 persons | 400 € |
|  |  | Families with 3 persons | 500 € |
|  |  | Families with 4 or more persons | 600 € |

**E-Kids incentive scale 1.1** Describes the number of food vouchers applicable to each participating family dependent on family members and corresponding stage participation. Food vouchers were give at the end of the study participation and good for two separate local grocery store chains.

## 1.3 E-Kids youth interview guideline

| Date | ID | Interviewer abbreviation | Beginning | End |
| --- | --- | --- | --- | --- |
|  |  |  |  |  |

|  | Key questions | Inquiries | Notes |
| --- | --- | --- | --- |
| ENTRY QUESTION | Can you describe what a typical food day looks like for you? | (using and referring to photo log) |  |
| EVERYDAY LIFE | - What does your school day look like in terms of food? - What do you eat when you're out with friends? | - School meals/ buy/ take with you on the way to school - Current trends? |  |
| HABITS, HOBBIES, PREFERENCES | - If you were to describe to someone what and how you eat, how would you describe it? - What is your favorite food? Why is this your favorite food? - What role do sport/exercise or other hobbies play in your everyday life and does this also have an influence on your diet? | - Type of diet (vegetarian/ halal/ high protein) - Influence of religion |  |
| FAMILY CONTEXT | - How are your family meals organized? - Who is responsible for shopping and cooking in your family? | - Are there certain meals that you always eat together? - Celebrations - Shopping procedure - Where do the ideas for what to cook come from? | - Question about ethnic background, if applicable |
| ADJUSTMENT | - How satisfied are you with your diet (scale 1-10) - What would you like to eat more/less of? | - Why is it that you don't eat more/less of it? - Why would you like to change that? |  |
| CONCLUSION | What other nutrition topics have I not covered that are important to you or your friends? |  |  |

**E-Kids youth interview guideline 1.3** The semi-structured question guideline for young participants

## 1.4 E-Kids expert interview guideline

| Date | ID | Interviewer abbreviation | Beginning | End |
| --- | --- | --- | --- | --- |
|  |  |  |  |  |

|  | Key question | Inquiries | Notes |
| --- | --- | --- | --- |
| PARTICIPATION ENCOURAGEMENT | How do you perceive the willingness of young people to participate in external projects like ours? | - Is there a specific situation that you can describe? - What could help to attract participants? |  |
| PERCEPTION OF EATING BEHAVIOR | What is your **perception of nutrition/eating behavior** among young people? | - What do you perceive as an influencing factor(s) in this regard? (social welfare, trends, peer group, etc.) - Can you describe the particular situation? |  |
| YOUTH WORK | How is the topic of **food/nutrition** addressed in your **work with young people**? | - Current projects - Utilization of meal offer - Difficulties arising from the topic of food/nutrition |  |
| CHANGE / INFLUENCE | What could bring about a **change in** young people's **eating behavior**? | - Are there certain places where food is offered (school meals, at home, friends) that have an important influence? - Do you have any influence on eating behavior in youth work? - Could you have more influence or who has the most influence on eating behavior? |  |
| END | What advice would you give to research projects like ours? |  |  |

**E-Kids expert interview guideline 1.4** The semi-structured questions for interviewed expert

## 1.5 E-kids participatory fieldnote form

**Observation protocol focused ethnography**

| Date | ID | Abbreviation employee | Start-End | Type of observation |
| --- | --- | --- | --- | --- |
|  |  |  |  | ☐︎ Purchasing ☐︎Mahlzeit |

1. **Summary Concise (one sentence)**

2. **Description Observation site**

**Drawing**

3. **Brief description of the process**

4. **Context Event (reason for the meeting)**

5. **Which persons were present (number, description, roles)**

6. **Precise and detailed description of the interactions**

7. **Description of food/dishes (preparation, presentation, etc.)**

8. **What were the most important topics/observations/emotions or what stood out the most?**

9. **What problems/conflicts have arisen?**

10. **Which aspects of the young people's perception of nutrition become clear in the given situation? ( dynamics, cultural background)**

11. **What should be considered in the future?**

12. **Do the observations in the field have certain methodological consequences?**

13. **Theoretical reflection**

14. **Reflecting on your own role/position in the situation**

15. **Free writing**

## 1.6 Parent Questionnaires

Parent Questionnaire — Nutrition

| **ID** | **EKJ01** | **EKJ02** | **EKJ03** | **EKJ04** | **EKJ05** | **EKJ06** | **EKJ07** | **EKJ08** | **Legend** |
| --- | --- | --- | --- | --- | --- | --- | --- | --- | --- |
| Question 1: How often do you cook yourself during the week using mostly fresh ingredients? | 2 |  |  |  | 2 |  |  |  | *0 = no answer; 1 = 2x/day or more; 2 = 1x/day or more; 3 = at least every 2 days; 4 = approx. 3x/week; 5 = < 3x/week; 6 = rarely to never* |
| Question 2: How often do you cook using ready-made / convenience products? | 6 |  |  |  | 4 |  |  |  | *0 = no answer; 1 = 2x/day or more; 2 = 1x/day or more; 3 = at least every 2 days; 4 = approx. 3x/week; 5 = < 3x/week; 6 = rarely to never* |
| Question 3: How often do you order food from outside / get takeaway from elsewhere? | 6 |  |  |  | 6 |  |  |  | *0 = no answer; 1 = 2x/day or more; 2 = 1x/day or more; 3 = at least every 2 days; 4 = approx. 3x/week; 5 = < 3x/week; 6 = rarely to never* |

Parent Questionnaire — Child Health and Finances

| **ID** | **EKJ01** | **EKJ02** | **EKJ03** | **EKJ04** | **EKJ05** | **EKJ06** | **EKJ07** | **EKJ08** | **Legend** |
| --- | --- | --- | --- | --- | --- | --- | --- | --- | --- |
| Question 1: How would you describe your child's state of health? | 1 |  |  |  | 2 |  |  |  | *0 = no answer; 1 = very good; 2 = good; 3 = fair; 4 = poor; 5 = very poor* |
| Question 2: How often do you check your bills for accuracy? | 5 |  |  |  | 5 |  |  |  | *0 = no answer; 1 = never; 2 = rarely; 3 = sometimes; 4 = mostly; 5 = always* |
| Question 3: How often do you pay your bills on time? | 4 |  |  |  | 5 |  |  |  | *0 = no answer; 1 = never; 2 = rarely; 3 = sometimes; 4 = mostly; 5 = always* |
| Question 4: How often do you pay your credit card bill in full in order to avoid interest costs? | 5 |  |  |  | 0 |  |  |  | *0 = no answer; 1 = never; 2 = rarely; 3 = sometimes; 4 = mostly; 5 = always; 6 = I do not have a credit card* |
| Question 5: How often do you use a spending plan or a budget? | 4 |  |  |  | 5 |  |  |  | *0 = no answer; 1 = never; 2 = rarely; 3 = sometimes; 4 = mostly; 5 = always* |
| Question 6: How often do you review your income and expenses before making larger purchases? | 4 |  |  |  | 5 |  |  |  | *0 = no answer; 1 = never; 2 = rarely; 3 = sometimes; 4 = mostly; 5 = always* |
| Question 7: How well are you able to manage the money available to you? | 2 |  |  |  | 1 |  |  |  | *0 = no answer; 1 = very good; 2 = good; 3 = fair; 4 = not so good; 5 = poor* |
| Question 8: How would you rate your competence in handling money? | 2 |  |  |  | 1 |  |  |  | *0 = no answer; 1 = very competent; 1 = competent; 2 = not so competent; not competent at all* |

# 2 Supplementary – Figures

## 2.1 E-kids – Photovoice

|   *Figure 1 – Photo Documentation: Breakfast at home (EKJ01)* |   *Figure 2 – Photo Documentation: After school snack (EKJ01)* |
| --- | --- |
|   *Figure 3 – Photo Documentation: DIY Flatbread for dinner (EKJ01)* |   *Figure 4 – Photo Documentation: Dinner Leftovers as evening snack (EKJ01)* |

*Figure 5 – Photo Documentation: Apple for a short day at school (EKJ01)*

*Figure 6 – Photo Documentation: Traditional snack for children (EKJ01)*

*Figure 7 – Photo Documentation: “Healthy” afternoon snack (EKJ01)*

*Figure 8 – Photo Documentation: Breakfast on the weekend (EKJ01)*

*Figure 9 – Photo Documentation: Study Snack (EKJ01)*

*Figure 10 – Photo Documentation: Fruit for whole family (EKJ01)*

*Figure 11 – Photo Documentation: Family recipe bean stew (EKJ01)*

*Figure 12 – Photo Documentation: Breakfast (EKJ01)*

*Figure 16 – Photo Documentation: Weekend Breakfast (EKJ02)*

*Figure 13 – Photo Documentation: Cake and fruit at the weekend (EKJ01)*

*Figure 14 – Photo Documentation: Weekly dinner (EKJ01)*

*Figure 15 – Photo Documentation: Weekend family dinner (EKJ01)*

*Figure 20 – Photo Documentation: favorite iced tea (EKJ02)*

*Figure 17 – Photo Documentation: School lunch (EKJ02)*

*Figure 18 – Photo Documentation: Pre-dinner salad (EKJ02)*

*Figure 19 – Photo Documentation: Sunday morning breakfast (EKJ02)*

*Figure 24 – Photo Documentation: dinner for kids (EKJ02)*

*Figure 23 – Photo Documentation: dinner for parents (EKJ02)*

*Figure 22 – Photo Documentation: salad as a snack (EKJ02)*

*Figure 21 – Photo Documentation: water bottle of the day (EKJ02)*

*Figure 27 – Photo Documentation: Dinner at restaurant with friends (EKJ04)*

*Figure 28 – Photo Documentation: Dinner at restaurant with friends, overview (EKJ04)*

*Figure 26 – Photo Documentation: Takis, a popular snack (EKJ02)*

*Figure 25 – Photo Documentation: Pasta with tomato sauce with fruit juice for dinner (EKJ02)*

*Figure 29 – Photo Documentation: small breakfast before school (EKJ05)*

*Figure 32 – Photo Documentation: breakfast on the way to school (EKJ05)*

*Figure 31 – Photo Documentation: muesli after school (EKJ05)*

*Figure 30 – Photo Documentation: lunch when mother is not at home yet (EKJ05)*

*Figure 36 – Photo Documentation: fruits and crisps for movie night with mother (EKJ05)*

*Figure 35 – Photo Documentation: favorite snack from international supermarket (EKJ05)*

*Figure 34 – Photo Documentation: pickled pre-dinner snack (EKJ05)*

*Figure 33 – Photo Documentation: lunch for participant and brother prepared by mother (EKJ05)*

*Figure 38 – Photo Documentation: biscuits that were on sale (EKJ05)*

*Figure 40 – Photo Documentation: late weekend breakfast (EKJ05)*

*Figure 39 – Photo Documentation: passion fruit in the afternoon (EKJ05)*

*Figure 37 – Photo Documentation: Tuesday dinner with the family (EKJ05)*

*Figure 42 – Photo Documentation: homemade dessert (EKJ05)*

*Figure 41 – Photo Documentation: ice cream as afternoon snack (EKJ05)*

*Figure 46 – Photo Documentation: Simit to eat during school break (EKJ06)*

*Figure 45 – Photo Documentation: Energy Drink during school break (EKJ06)*

*Figure 44 – Photo Documentation: Sunday breakfast with parents (EKJ06)*

*Figure 43 – Photo Documentation: Saturday breakfast with parents (EKJ06)*

*Figure 50 – Photo Documentation: candy floss at the fair (EKJ06)*

*Figure 48 – Photo Documentation: grilled sausage at the fair (EKJ06)*

*Figure 49 – Photo Documentation: homemade cake by participant and mother (EKJ06)*

*Figure 47 – Photo Documentation: crisps, described as favorite snack (EKJ06)*

*Figure 54 – Photo Documentation: eating at home with the family (EKJ06)*

*Figure 53 – Photo Documentation: snacking while studying (EKJ06)*

*Figure 52 – Photo Documentation: sweets of the week (EKJ06)*

*Figure 51 – Photo Documentation: grapes giving by mother (EKJ06)*

*Figure 58 – Photo Documentation: lunch on the go (EKJ06)*

*Figure 57 – Photo Documentation: typical weekend breakfast (EKJ06)*

*Figure 56 – Photo Documentation: lunch when home alone (EKJ06)*

*Figure 55 – Photo Documentation: quick breakfast before school (EKJ06)*

*Figure 60 – Photo Documentation: typical dinner on the weekend (EKJ07)*

*Figure 59 – Photo Documentation: homemade Baklava (EKJ06)*

**E-Kids photovoice.** Photographs made by participating young people from their consumed food, corresponding description and participant
